# Supplementary material for: A solvent-assisted ligand exchange approach enables metal-organic frameworks with diverse and complex architectures
Source: Nat Commun. 2020 Feb 17;11:927. doi: 10.1038/s41467-020-14671-9 (PMC7026438; doi:10.1038/s41467-020-14671-9)
Supplement: Supplementary file 1 — Supplementary Information [file 41467_2020_14671_MOESM1_ESM.pdf]

Supplementary Information

A solvent-assisted ligand exchange approach enables metal-organic frameworks with diverse and complex architectures

Yu et al.

Table of Contents

Supplementary Figure 1 Overview diagram of prepared MOF architectures..1

Supplementary Figure 2 Characterization of MOF-5-derived SH-ZIF-8..2

Supplementary Figure 3 FT-IR spectra of ZIF-based nanostructured materials.................................. 3

Supplementary Figure 4 Characterization of MOF-5@ZIF-8 yolk-shell nanocubes............................ 5

Supplementary Figure 5 Characterization of MOF-5-derived DH-ZIF-8.. 6

Supplementary Figure 6 Characterization of the resulting materials when MOF-5 in Hmim ethanol solution as a function of time..7

Supplementary Figure 7 SEM images of broken MOF-5-derived hollow ZIF-8 nanocubes................. 9

Supplementary Figure 8 SEM images of the resulting yolk-shell structures when MOF-5 in Hmim ethanol solution under different conditions.. 10

Supplementary Figure 9 Characterization of MOF-5-derived ball-in-box ZIF-8 nanocubes...............11

Supplementary Figure 10 SEM images of resulting materials when MOF-5 in Hmim ethanol solution as a function of concentration..12

Supplementary Figure 11 SEM images of the resulting products when MOF-5 in different Hmim solvent solutions... 13

Supplementary Figure 12 SEM images of the resulting product of ZnCo-MOF-5 in Hmim ethanol solution under different conditions..14

Supplementary Figure 13 Characterization of ZnCo-MOF-5-derived single-shelled hollow ZnCo-ZIF. 15

Supplementary Figure 14 Characterization of ZnCo-MOF-5-derived double-shelled hollow ZnCo-ZIF...16

Supplementary Figure 15 Characterization of ZIF-7-derived single-shelled hollow polyhedral ZIF-8. 17

Supplementary Figure 16 Characterization of ZIF-71-derived single-shelled hollow polyhedral ZIF-8. 18

Supplementary Figure 17 Characterization of ZIF-71-derived double-shelled hollow polyhedral ZIF-8. 19

Supplementary Figure 18 SEM images of the resulting materials when Zn-HMT nanosheets were added into different Hmim solvent solutions...20

Supplementary Figure 19 Characterization of Zn-HMT-derived ZIF-8 nanosheets........................... 21

Supplementary Figure 20 Characterization of Zn-HMT-derived sesame pancake-like Zn-HMT@ZIF-8 nanosheets..22

Supplementary Figure 21 SEM images of ZnCo-PPF-3 before and after being placed in 1 M Hmim solution within different solvents and reaction time..23

Supplementary Figure 22 Characterization of Zn-BTC-derived double-shelled ZIF-8 nanotubes...... 25

Supplementary Figure 23 Characterization of 90Zn10Co-BTC-derived double-shelled ZnCo-ZIF nanotubes.. 26

Supplementary Figure 24 TEM images of double-shelled and triple-shelled ZnCo-ZIF nanotubes....27

Supplementary Figure 25 Characterization of 50Zn50Co-BTC-derived single-shelled ZnCo-ZIF nanotubes.. 28

Supplementary Figure 26 Characterization of 90Zn10Co-BTC-derived bead-on-string structured 90Zn10Co-BTC@ZnCo-ZIF.. 29

Supplementary Figure 27 Characterization of 90Zn10Co-BTC-derived 90Zn10Co-BTC@ZnCo-ZIF core-shell structure....30
Supplementary Figure 28 Characterization of 90Zn10Co-BTC-derived 90Zn10Co-BTC@ZnCo-ZIF nanowire-nanotube structure....31
Supplementary Figure 29 Characterization of peapod-like ZnCo-ZIF....32
Supplementary Figure 30 TEM images of the final products by a traditional two-step synthesis....33
Supplementary Figure 31 Characterization of 20Zn80Co-MOF-74-derived core-shell structured 20Zn80Co-MOF-74@ZnCo-ZIF....34
Supplementary Figure 32 Characterization of 20Zn80Co-MOF-74-derived single-shelled ZnCo-ZIF nanotubes....35
Supplementary Figure 33 SEM images of 10Zn90Co-MOF-74 nanowires in different concentration of Hmim....36
Supplementary Figure 34 Characterization of ZIF-8 particles before and after the carbonization treatment....37
Supplementary Figure 35 XRD patterns of s-Zn-ZIF-C, DH-Zn-ZIF-C, NS-Zn-ZIF-C, DT-Zn-ZIF-C and DT-ZnCo-ZIF-C....38
Supplementary Figure 36 TEM-EDX mapping images of DH-Zn-ZIF-C, NS-Zn-ZIF-C and DT-Zn-ZIF-C....39
Supplementary Figure 37 The high-resolution N 1s XPS spectra and Raman spectra of different ZIF-derived porous carbon....40
Supplementary Figure 38 Nitrogen adsorption-desorption isotherms and pore size distribution plots of different ZIF-derived porous carbon....41
Supplementary Figure 39 CV plots of s-Zn-ZIF-C and DT-Zn-ZIF-C at different scan rates....42
Supplementary Figure 40 Contribution ratio of DIP and SCP versus scan rate for s-Zn-ZIF-C, DH-Zn-ZIF-C, NS-Zn-ZIF-C and DT-Zn-ZIF-C....43
Supplementary Figure 41 Nyquist plots of s-Zn-ZIF-C, DH-Zn-ZIF-C, NS-Zn-ZIF-C, DT-Zn-ZIF-C and DT-ZnCo-ZIF-C....44
Supplementary Table 1 Overall N-doping content of ZIF-derived porous carbon and the specific content of pyridinic N, pyrrolic N and graphitic N....45
Supplementary Table 2 Na^+ storage properties of MOF-derived carbon materials in recent literature. 46
Supplementary Table 3 Na^+ storage properties of other carbon materials in recent literature....48
Supplementary references....50

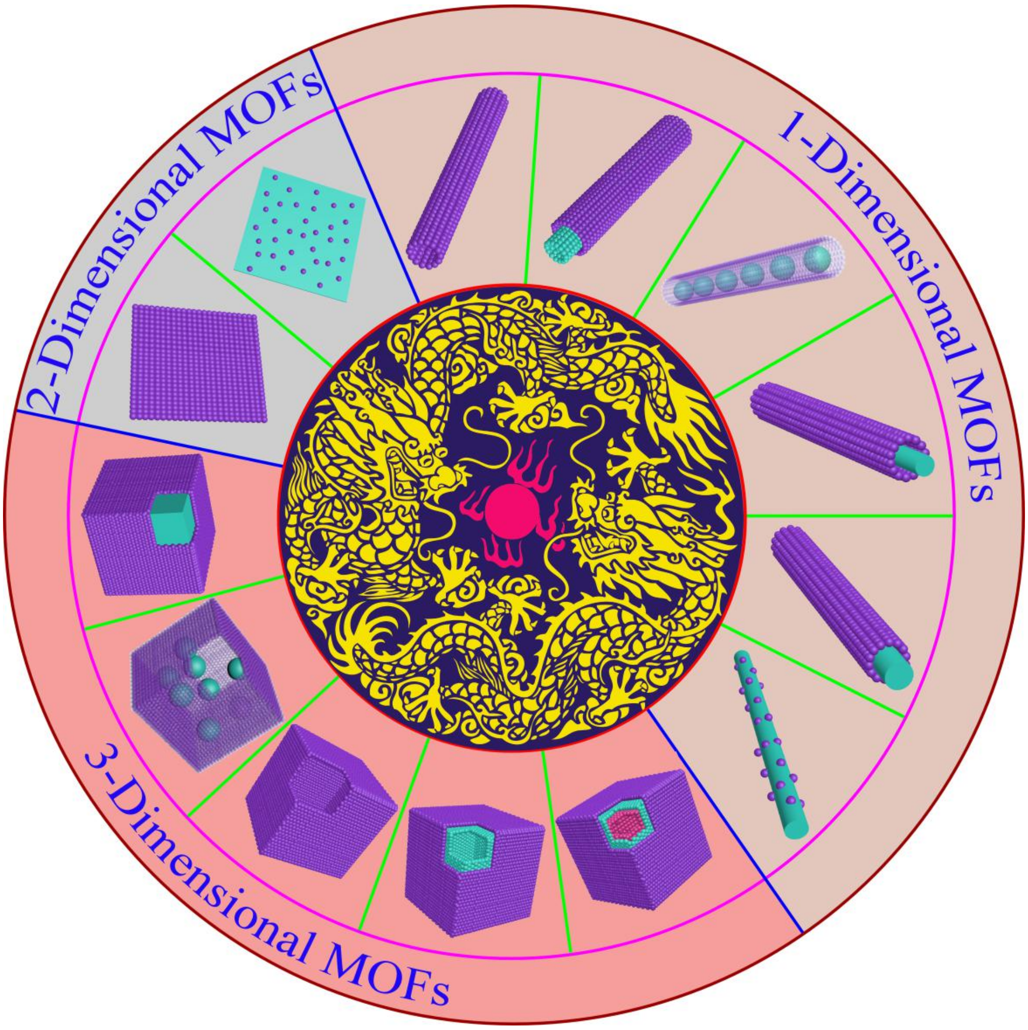

Supplementary Figure 1. Overview diagram of 13 different types of MOF nanostructures ranging from 3D to 1D and 2D via the solvent-assisted ligand exchange (SALE) strategy, with the "double dragon playing a ball" symbol (blue ring region) representing the SALE process. One Chinese loong corresponds to the cleavage of old coordination bonds (dissolution of mother MOFs), the other corresponds to the establishment of new coordination bonds (recrystallization of daughter MOFs), and the auspicious dragon ball symbolizes the 13 MOF nanoarchitectures achieved by our proposed approach.

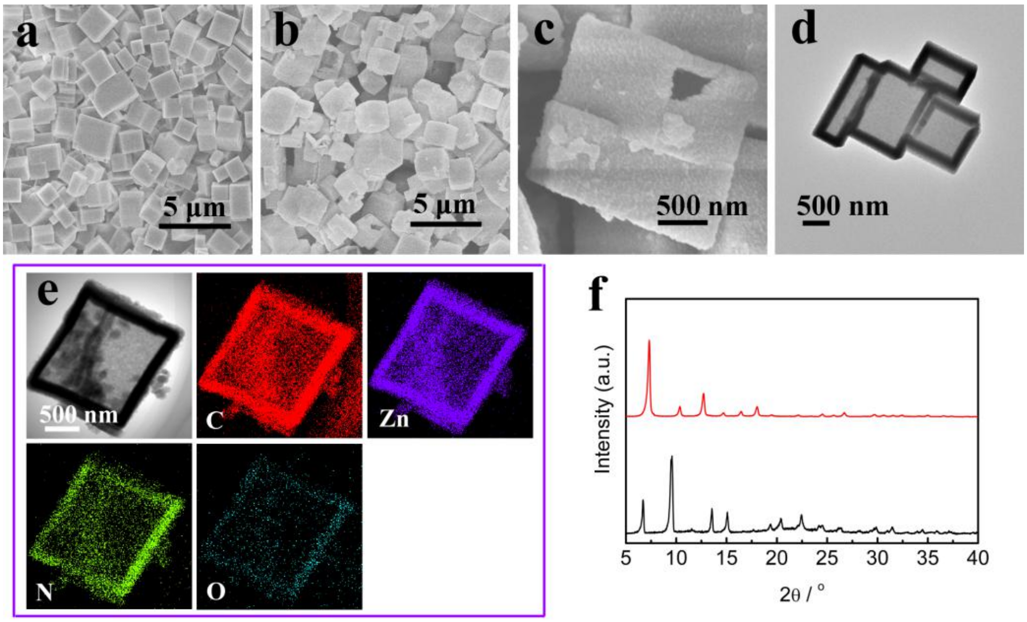

Supplementary Figure 2. SEM images of as-prepared MOF-5 nanocubes (a) and MOF-5-derived single-shelled hollow ZIF-8 nanocubes (SH-ZIF-8) (b, c); TEM image of SH-ZIF-8 (d); EDX mapping images of SH-ZIF-8 (e); XRD patterns of materials before (black line) and after (red line) the reaction; the peaks of the red line all corresponded to ZIF-8 (f), indicating the transformation from MOF-5 to SH-ZIF-8.

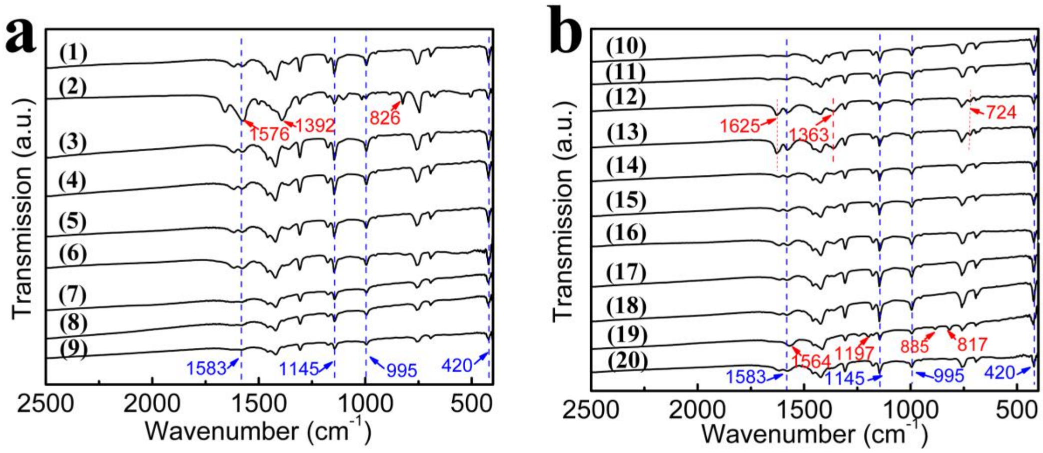

Supplementary Figure 3. FT-IR spectra of ZIF-based nanostructured materials: single-shelled hollow ZIF-8 nanocubes (1); MOF-5@ZIF-8 yolk-shell nanocubes (2); double-shelled hollow (3) and ball-in-box ZIF-8 nanocubes (4); single-shelled (5) and double-/triple-shelled (6) hollow ZnCo-ZIF nanocubes; ZIF-7-derived hollow ZIF-8 (7); ZIF-71-derived single-shelled (8) and double-shelled (9) hollow ZIF-8; ZIF-8 nanosheets (10); sesame pancake-like Zn-HMT@ZIF-8 nanosheets (11); bead-on-string (12), core-shell (13) and nanowire-nanotube (14) structured 90Zn10Co-BTC@ZnCo-ZIF; double-shelled ZIF-8 nanotubes (15); single-shelled (16) and double-shelled (17) ZnCo-ZIF nanotubes; peapod-like ZnCo-ZIF (18); ZnCo-MOF-74-derived core-shell nanowire (19) and single-shelled nanotube (20). The peak at 1583 cm^{-1} was assigned to the C=N stretch mode, the bands at 1145 and 995 cm^{-1} were associated with the C-N stretching, and the peak located at 420 cm^{-1} indicated the Zn/Co-N stretching. The bands at 1576, 1392 and 826 cm^{-1} corresponded to the asymmetric stretching, symmetric stretching and out-of-plane bending of aromatic C-H of BDC, respectively. The absorptions at 1625 and 1363 cm^{-1} were due to the presence of water and the symmetric carbonyl stretching vibrations of BTC^{3-} after coordination to Zn^{2+}/Co^{2+}, and the peak at 724 cm^{-1} was attributed to the 1,3,5-trisubstituted benzene. The features at 1564 and 1197 cm^{-1} could be ascribed to C=O and C-O stretching band, and the peaks appeared at 885 and 817 cm^{-1} were due to the C-H wagging vibration in and out plane of benzene ring. The FT-IR results of all prepared ZIF-based nanostructures clearly demonstrated the

coordination of 2-methylimidazole to Zn/Co, suggesting the transformation of ZIFs from those mother MOFs.

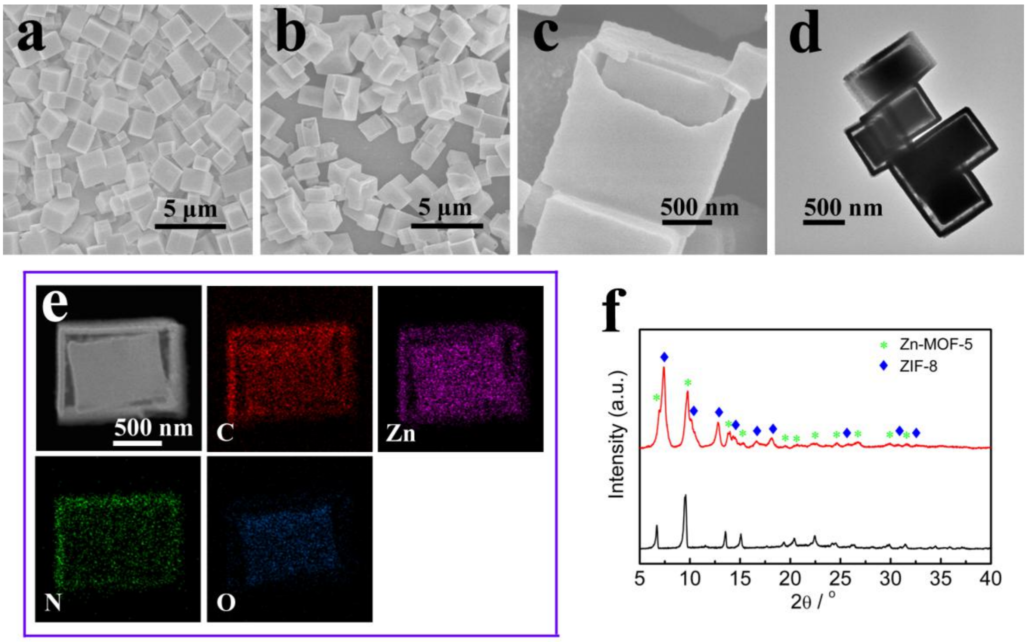

Supplementary Figure 4. SEM images of as-prepared MOF-5 nanocubes (a) and MOF-5@ZIF-8 yolk-shell nanocubes (b, c); TEM image of MOF-5@ZIF-8 yolk-shell nanocubes (d); EDX mapping images of MOF-5@ZIF-8 yolk-shell structure (e); the O and N element distribution revealed the yolk of MOF-5 and the shell of ZIF-8, respectively; XRD patterns of materials before (black line) and after (red line) the reaction; the XRD results indicated the partial transformation from MOF-5 to ZIF-8 (f), suggesting the yolk-shell structured MOF-5@ZIF-8 nanocubes. Interestingly, MOF-5 within this hybrid nanostructure was more stable than pristine MOF-5, and distinct diffraction peaks of MOF-5 were observed even when the sample was exposed in air for one week, almost no impurity peak could be detected. A possible explanation for this phenomenon was that the MOF-5 yolk may be decorated by a very thin ZIF-8 layer (stable and hydrophobic), which served as a protective cover to prevent the entrance of H_2O from dissociating MOF-5.

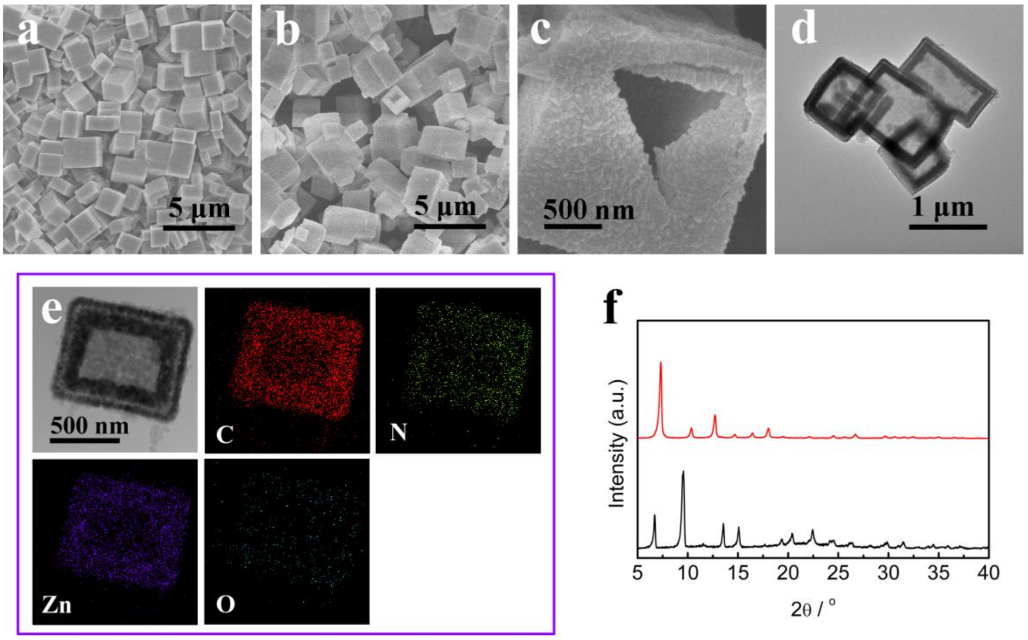

Supplementary Figure 5. SEM images of as-prepared MOF-5 nanocubes (a) and MOF-5-derived double-shelled hollow ZIF-8 nanocubes (DH-ZIF-8) (b, c); TEM image of DH-ZIF-8 (d); EDX mapping images of DH-ZIF-8 (e); XRD patterns of materials before (black line) and after (red line) the reaction; the peaks of the red line all corresponded to ZIF-8 and no other peak was detected (f), indicating the transformation from MOF-5 to DH-ZIF-8.

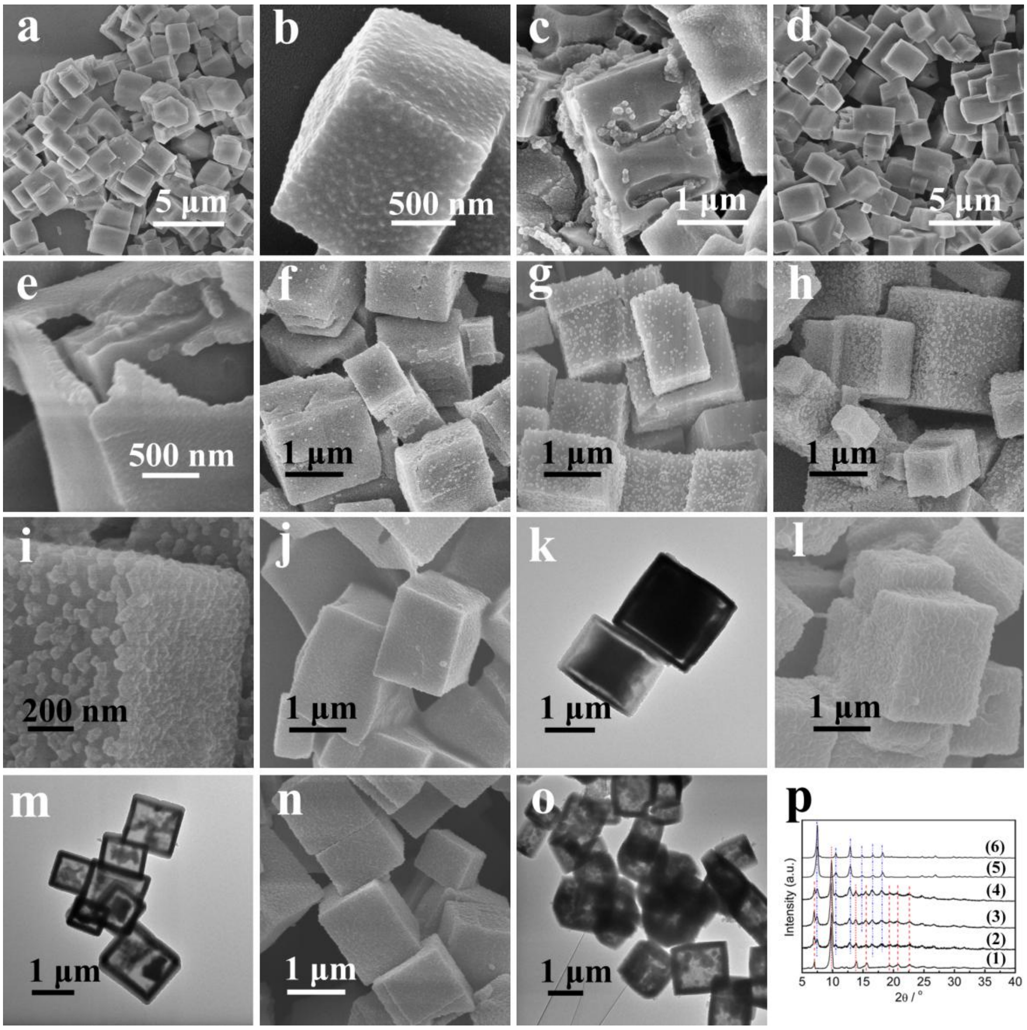

Supplementary Figure 6. SEM images of the resulting materials when MOF-5 was placed in Hmim ethanol solution: 1.2 M Hmim for 10 s (a-c) and 30 s (d-e); 0.3 M Hmim for 10 s (f), 30 s (g), 2 min (h-i), 10 min (j), 1 h (l) and 3 h (n). TEM images of the resulting materials when MOF-5 was placed in 1.2 M Hmim for 10 min (k), 1 h (m) and 3 h (o). XRD patterns of the materials obtained when MOF-5 was placed in 0.3 M Hmim at different time (p): 10 s (1), 30 s (2), 2 min (3), 10 min (4), 1 h (5) and 3 h (6). When experiments were conducted in 1.2 M Hmim solution, as the transformation proceeded for 10 s, small humps could be observed on the surface of nanocubes (b), the enlarged image of broken nanocubes revealed that a ZIF-8 layer had compactly grown on the surface of MOF-5 to produce a core-shell hybrid structure (c); prolonging time to 20 s, phase separation between MOF-5 and ZIF-8 happened, and a yolk-shell structure was obtained (e), suggesting the rapid accumulation of vacancies at the phase boundary within a short time due to much

faster dissolution of MOF-5 (cleavage of Zn-O bond) than recrystallization of ZIF-8 (establishment of Zn-N bond). In order to get more detailed information of the transformation as a function of time, 0.3 M Hmim was used to slow down the transformation kinetics. We could clearly see that small nanocrystals were formed on the surface of MOF-5 in 10 s (f), the nanocrystal number gradually increased with the time (g-i), and MOF-5 facets were completely covered by nanocrystals (i); the XRD results indicated the presence of ZIF-8 phase, confirming the conversion of ZIF-8 from MOF-5 (p). Yolk-shell structure emerged at 10 min (k), the limited inward diffusion of mim^- enabled the fast dissolution of MOF-5 but sluggish crystallization of ZIF-8 (l-o); the ZIF-8 crystallization would not start until the ratio of diffused mim^- to dissociated Zn^{2+} reached a certain value, secondary ZIF-8 shell could not evolve from the yolk MOF-5, and it finally generated a ball-in-box structure under such preparation condition (o). In Supplementary Figure 1, single-shelled ZIF-8 hollow nanocubes were synthesized in 1.2 M Hmim solution as ZIF-8 continuously grew on the inner wall of ZIF-8 shell, resulting from the trend of reducing nucleation energy and relatively higher mim^-/Zn^{2+} ratio nearby the inner wall. When dilute Hmim solution (0.3 M) was used, it took time to achieve sufficient mim^-/Zn^{2+} ratio for crystallizing ZIF-8 inside the enclosed shell while MOF-5 was quickly dissolved, and the very low mim^-/Zn^{2+} ratio induced the growth of big ZIF-8 nanoparticles. We could also assume that big ZIF-8 nanoparticles possibly crystallized on the inner wall of shell, but the active nucleation sites were limited, the contact between the shell wall and big particles was too weak, these big ZIF-8 nanoparticles would fall off from the inner wall and thus form the "ball" of ball-in-box structure.

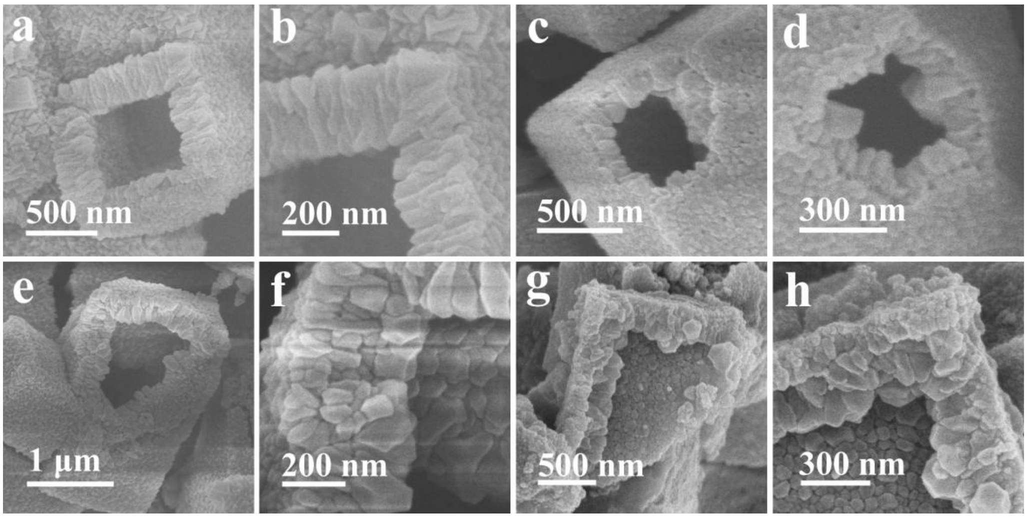

Supplementary Figure 7. SEM images of broken MOF-5-derived ZIF-8 nanocubes: SH-ZIF-8 (a, b); DH-ZIF-8 (c-h); b, d, f and h are the corresponding enlarged images of a, c, e and g, respectively. Through carefully examining the cross-section walls of these broken nanocubes, we could clearly observe that the walls were composed of numerous columnar crystals, very similar to the columnar grain zone in a casting-state structure. This phenomenon could be explained by the classic competitive growth mechanism: once an ultrathin ZIF-8 layer was formed, the ion/molecule transfer was restricted, and the further growth of ZIF-8 was diffusion-controlled; because only a smaller number of mim^- ligands was transported through the ZIF-8 layer, forming a descending ligand concentration gradient from outside to inside (compared to a descending concentration gradient of temperature in the casting-state structure), the kinetics of heterogeneous nucleation was comparatively lower, which could rightly overcome the energy barrier of highly preferred orientation. As a result, ZIF-8 followed the oriented growth. It was also observed that the particle size of ZIF-8 gradually increased from the outer shell to the inner shells (c-h), originating from the increasing molar ratio of Zn^{2+}/mim^-, which further confirmed the diffusion-control effect.

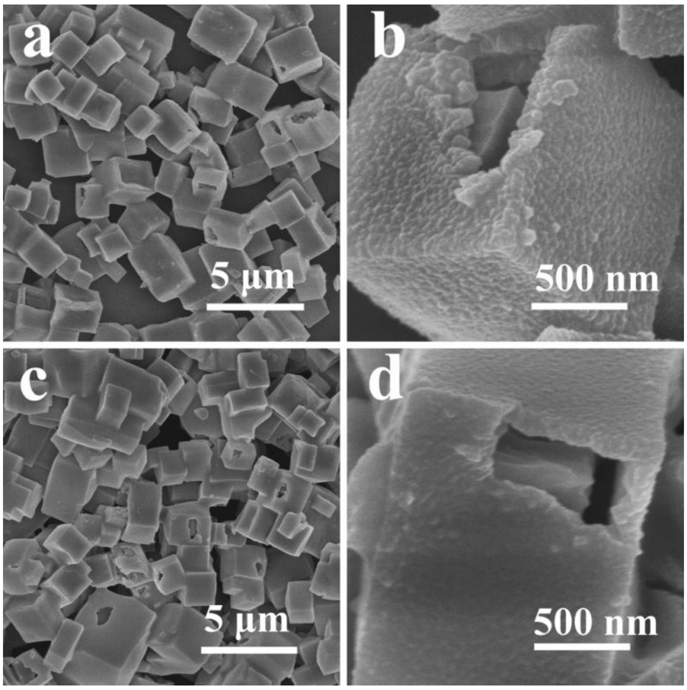

Supplementary Figure 8. SEM images of the resulting yolk-shell structures when MOF-5 was placed in Hmim ethanol solution: 1.2 M Hmim solution at 50 ^{o}C for 30 s (a, b); 0.6 M Hmim solution at room temperature for 10 min (c, d). Combined with Supplementary Figures 4-6, it could be concluded that the yolk-shell structure was an intermediate state for the SH-ZIF-8, DH-ZIF-8 and ball-in-box ZIF-8 structures.

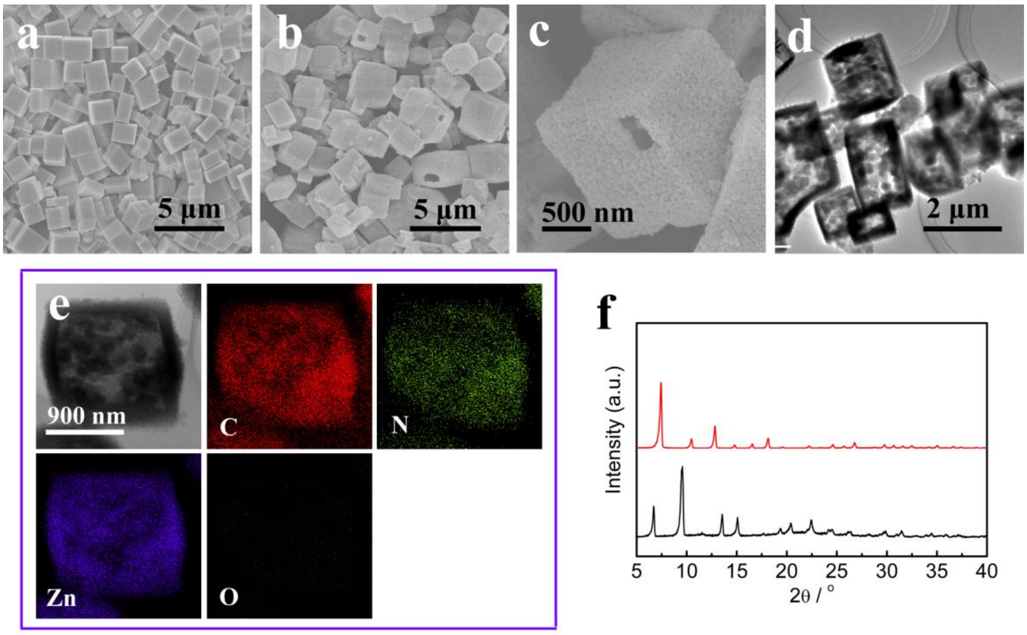

Supplementary Figure 9. SEM images of as-prepared MOF-5 nanocubes (a) and MOF-5-derived ball-in-box ZIF-8 nanocubes (b, c); TEM image of ball-in-box ZIF-8 (d); EDX mapping images of ball-in-box ZIF-8 (e); XRD patterns of materials before (black line) and after (red line) the reaction; the peaks of the red line all corresponded to ZIF-8, and no other peak was detected (f), indicating the transformation from MOF-5 to ball-in-box ZIF-8.

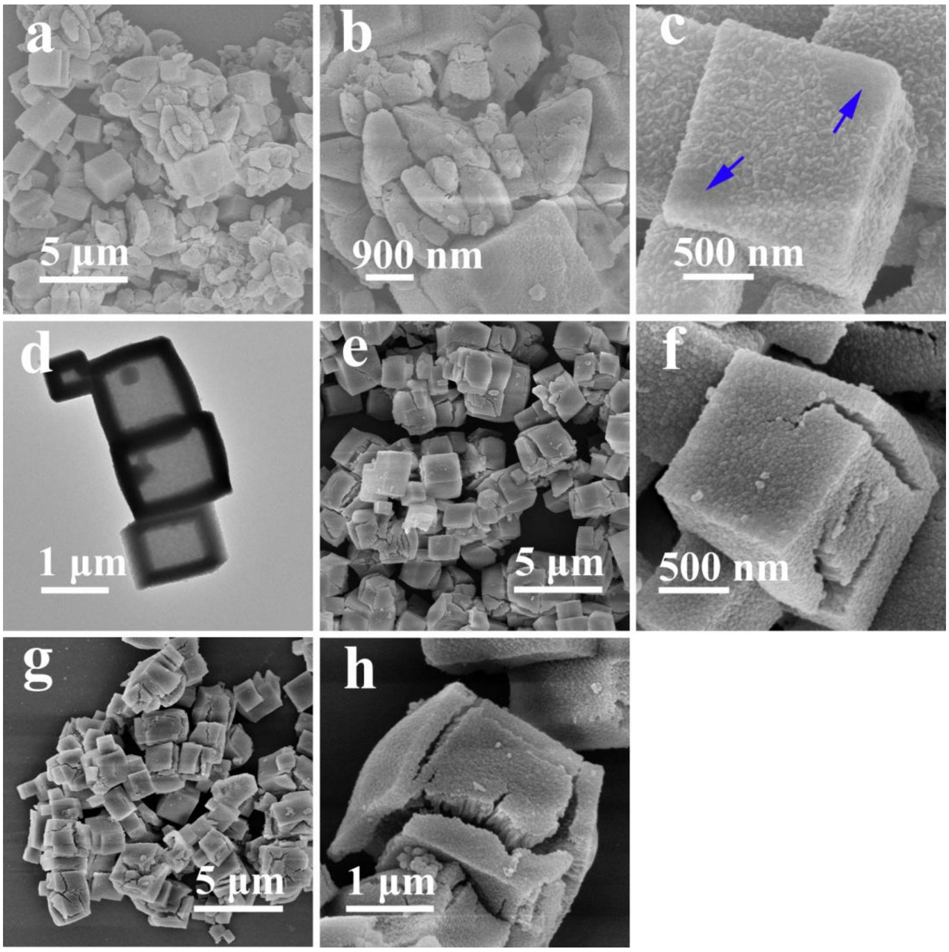

Supplementary Figure 10. SEM images of resulting materials when MOF-5 was placed in Hmim ethanol solution overnight at different concentrations: 0.15 M (a-d), 2.4 M (e-f) and 4.8 M (g-h). At 0.15 M, the product was not a homogeneous material but a mixture (a); bulks without cubic shape were most likely metastable Zn-Hmim/Zn-BDC coordination compounds before forming ZIF-8 (b), resulting from the insufficient Hmim[1]; ZIF-8 nanocrystals had never covered all the surfaces of some nanocubes (c), implying the incomplete conversion to ZIF-8; it seemed like all of nanocubes had hollow feature (d), the formation mechanism might differ from that of SH-ZIF-8, phase separation would never happen because the created vacancies had enough time to diffuse into the center rather than accumulate at the phase interface, as low concentration enabled slow kinetics. At very high concentrations, large cracks appeared due to the stress concentration during the violent transformation process (e-h).

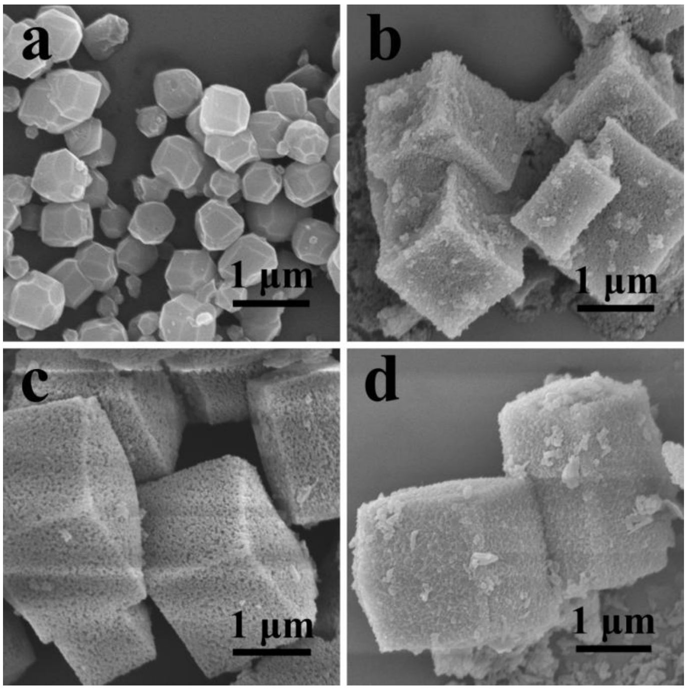

Supplementary Figure 11. SEM images of the resulting products when MOF-5 was placed in different Hmim solvent solutions: water (a), methanol (b), N,N-dimethylformamide (c) and N-methyl pyrrolidone (d). We could see that by replacing water as the solvent, monodispersed ZIF-8 particles were produced (a); by contrast, there was no large difference in the final materials when ethanol, methanol, N,N-dimethylformamide or N-methyl pyrrolidone was used as the solvent because water enabled the strongest deprotonation of Hmim, which caused a dissolution rate that was so fast that no substrate was available for nucleation of ZIF-8.

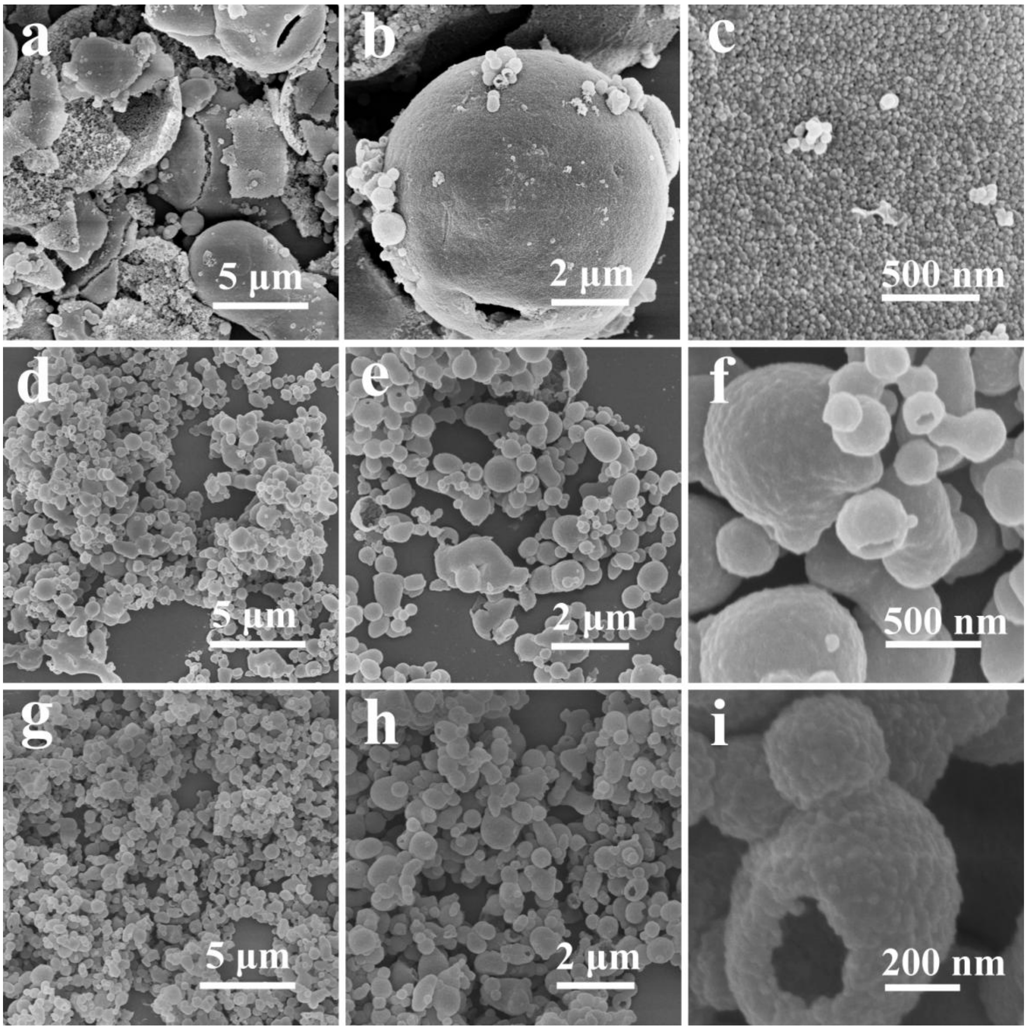

Supplementary Figure 12. SEM images of the resulting product of ZnCo-MOF-5 at 45 ^{o}C: in 1.2 M (a-c), 0.3 M (d-f) and 0.15 M (g-i) Hmim ethanol solution. All the products lost the initial shape of mother ZnCo-MOF-5 because the cleavage of Zn-/Co-BDC was much faster than that of Zn-/Co-mim, the recrystallization of ZnCo-ZIF was not able to occur on the surface of ZnCo-MOF-5 and consume the dissociated Zn^{2+}/Co^{2+} ions.

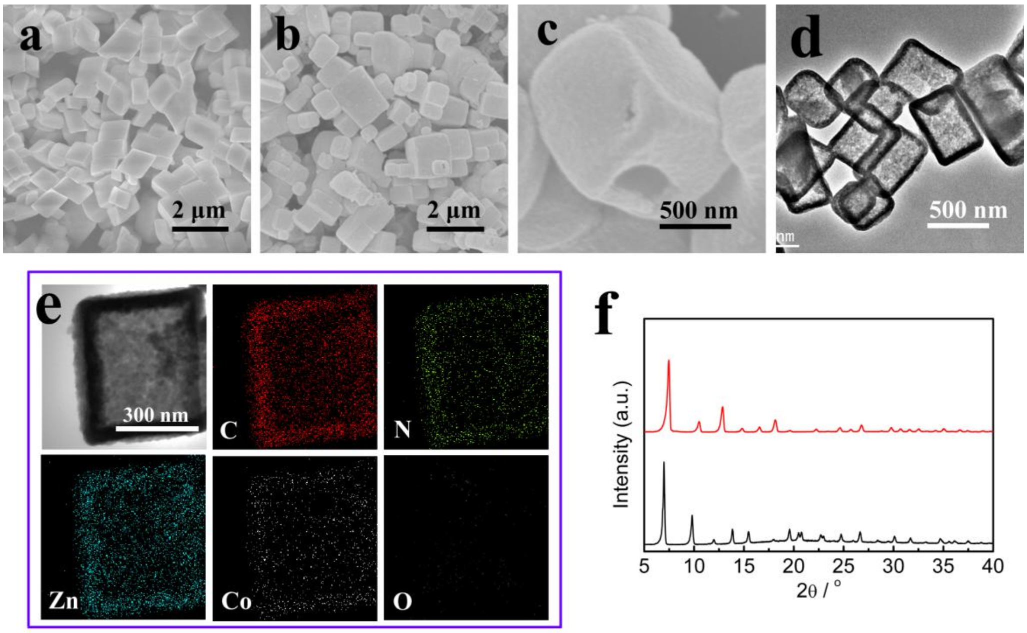

Supplementary Figure 13. SEM images of as-prepared Co-doped MOF-5 (a) and its derived single-shelled hollow ZnCo-ZIF (b, c); TEM image of single-shelled hollow ZnCo-ZIF (d); EDX mapping images of single-shelled hollow ZnCo-ZIF (e); XRD patterns of materials before (black line) and after (red line) the reaction (f); the peaks of the red line all corresponded to ZnCo-ZIF, and no other peak was detected, indicating the transformation from ZnCo-MOF-5 to ZnCo-ZIF. The molar ratio of Zn^{2+}:Co^{2+} for preparing Co-doped MOF-5 was 9:1.

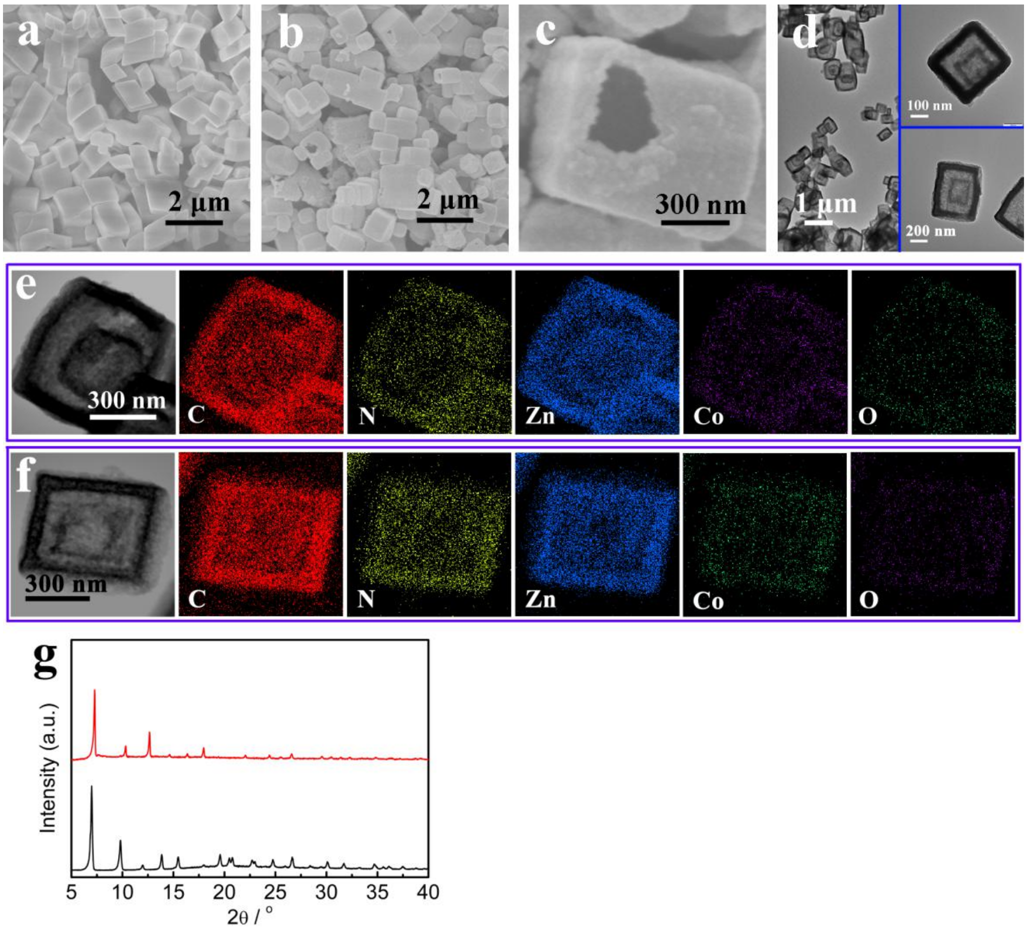

Supplementary Figure 14. SEM images of as-prepared Co-doped MOF-5 (a) and its derived double-shelled hollow ZnCo-ZIF (b, c); TEM images of double-shelled and triple-shelled hollow ZnCo-ZIF (d), triple-shelled hollow ZnCo-ZIF held 5% ~ 10% of the final product according to TEM observation; EDX mapping images of double-shelled hollow ZnCo-ZIF (e) and triple-shelled hollow ZnCo-ZIF (f); XRD patterns of materials before (black line) and after (red line) the reaction, the peaks of the red line all corresponded to ZnCo-ZIF, and no other peak was detected (g), indicating the transformation from ZnCo-MOF-5 to ZnCo-ZIF. The molar ratio of Zn^{2+}/Co^{2+} for preparing Co-doped MOF-5 was 19:1. It was seen that the gap between outer shell and inner shell for double-shelled hollow ZnCo-ZIF was much larger than for MOF-5-derived DH-ZIF-8, and the thickness of the inner shell was thinner, which could be ascribed to the low recrystallization rate of ZnCo-ZIF.

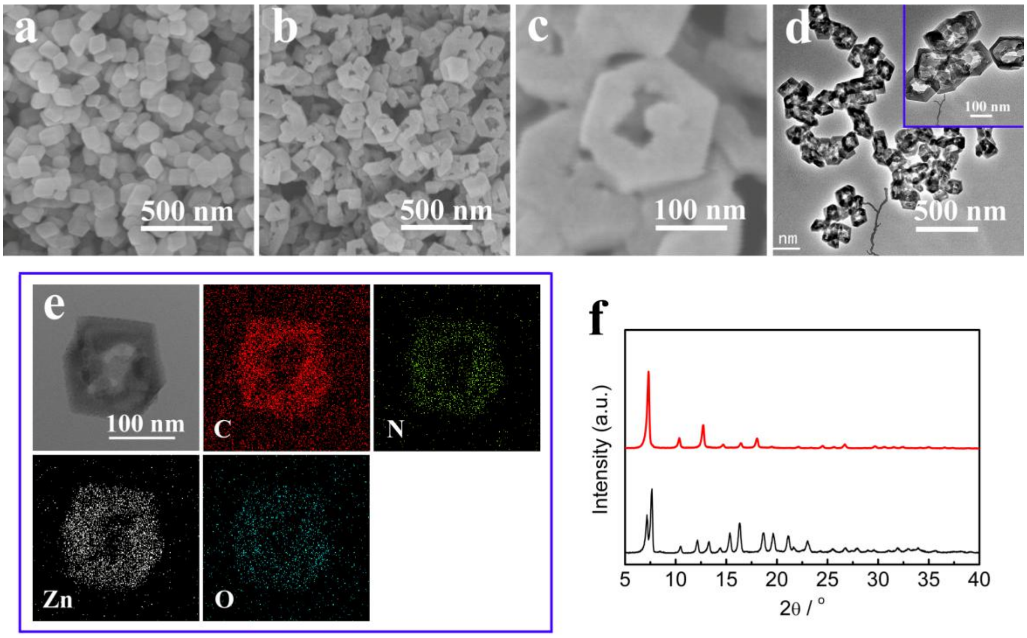

Supplementary Figure 15. SEM images of as-prepared ZIF-7 (a) and ZIF-7-derived single-shelled hollow polyhedral ZIF-8 (b, c); TEM image of single-shelled hollow ZIF-8 (d); EDX mapping images of single-shelled hollow ZIF-8 (e); XRD patterns of materials before (black line) and after (red line) the reaction (f); the peaks of the red line all corresponded to ZIF-8, and no other peak was detected, indicating the transformation from ZIF-7 to ZIF-8. This transformation was conducted at 45 ^{o}C in 2.4 M ethanol solution, and even if the particle size of ZIF-7 was only ~ 100 nm, its derived ZIF-8 had a single-shelled hollow structure; in contrast, under the same reaction conditions, the ZIF-71-derived product was double-shelled hollow ZIF-8, confirming the higher stability of ZIF-7 than of ZIF-71.

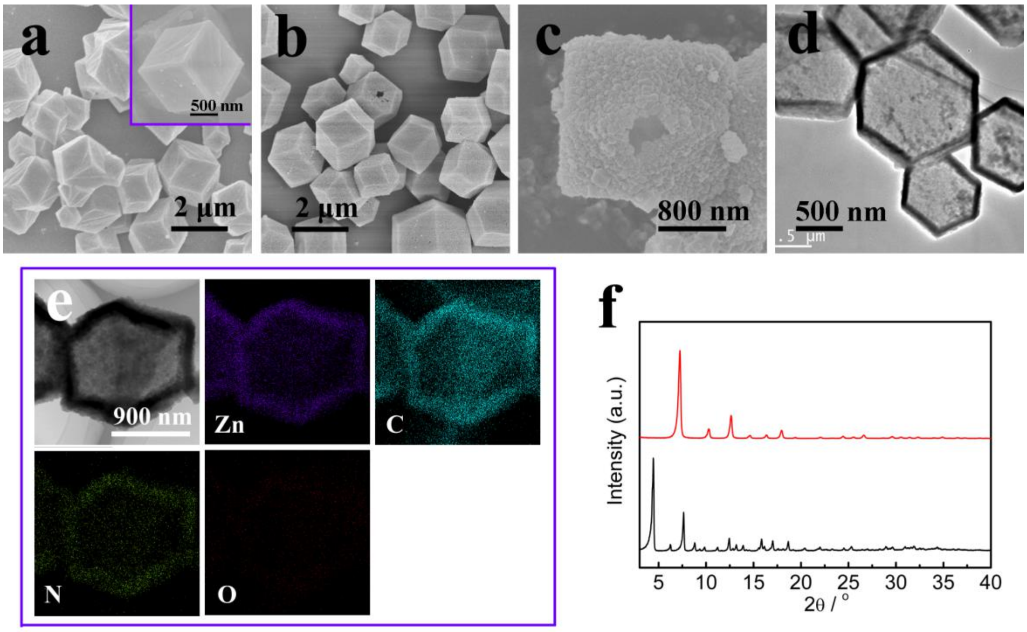

Supplementary Figure 16. SEM images of as-prepared ZIF-71 (a) and ZIF-71-derived single-shelled hollow polyhedral ZIF-8 (b, c); TEM image of single-shelled hollow ZIF-8 (d); EDX mapping images of single-shelled hollow ZIF-8 (e); XRD patterns of materials before (black line) and after (red line) the reaction (f); the peaks of the red line all corresponded to ZIF-8, and no other peak was detected, indicating the transformation from ZIF-71 to ZIF-8. This transformation was conducted at 45 °C in 1.2 M ethanol solution. Under the same reaction conditions, because the fast accumulation of vacancy at the MOF-5/ZIF-8 phase interface due to a non-equivalent diffusion effect, phase separation would happen; the MOF-5-derived product was double-shelled hollow ZIF-8, confirming the higher stability of ZIF-71 than that of MOF-5.

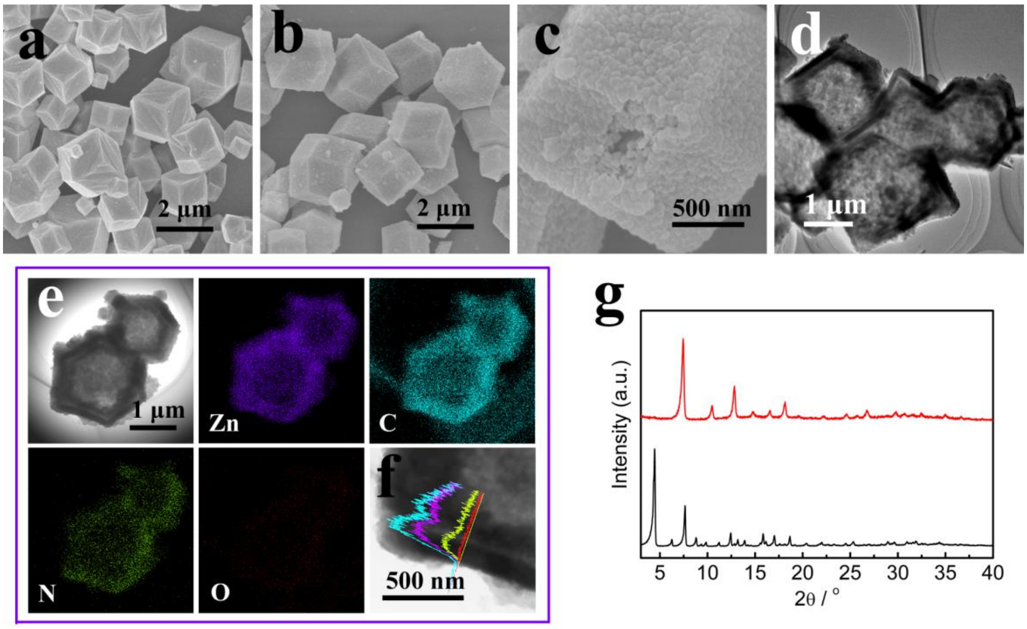

Supplementary Figure 17. SEM images of as-prepared ZIF-71 (a) and ZIF-71-derived double-shelled hollow ZIF-8 polyhedron (b, c); TEM image of double-shelled hollow ZIF-8 (d); EDX mapping images (e) and linear scanning image (f) of double-shelled hollow ZIF-8; XRD patterns of materials before (black line) and after (red line) the reaction (g); the peaks of the red line all corresponded to ZIF-8, and no other peak was detected, indicating the transformation from ZIF-71 to ZIF-8. The higher mim^- concentration in this case would enable the sufficient mim- ligands inside the hollow interior for crystallizing ZIF-8 on the surface of internal yolk ZIF-71 immediately, leading to the formation of secondary ZIF-8 shell. The thickness of the inner shell was almost as thick as that of the outer shell as a result of the mild SALE process between ZIF-8 and ZIF-71. This transformation was conducted at 45 ^{o}C in 2.4 M ethanol solution, under the same reaction conditions, the ZIF-7-derived product was single-shelled hollow ZIF-8, confirming the lower stability of ZIF-71 than of ZIF-7.

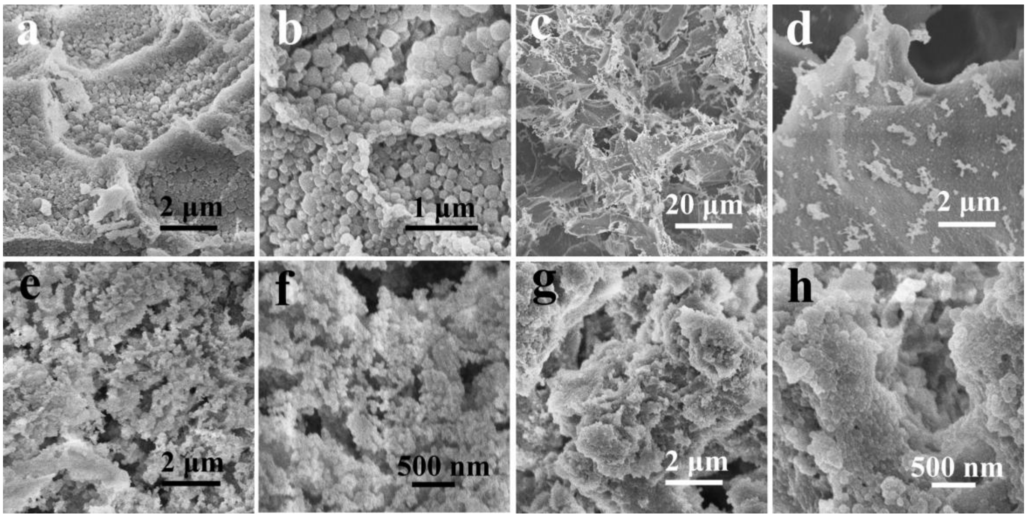

Supplementary Figure 18. SEM images of the resulting materials when Zn-HMT nanosheets were added into a 1 M Hmim solution: water (a, b), ethanol (c, d), N,N-dimethylformamide (e, f) and N-methyl pyrrolidone (g, h) as the solvents. If methanol was replaced by other common solvents such as water, ethanol, N,N-dimethylformamide and N-methyl pyrrolidone, the final materials all lost the initial nanosheet morphology. In addition, only very little product could be collected in the case of N,N-dimethylformamide or N-methyl pyrrolidone.

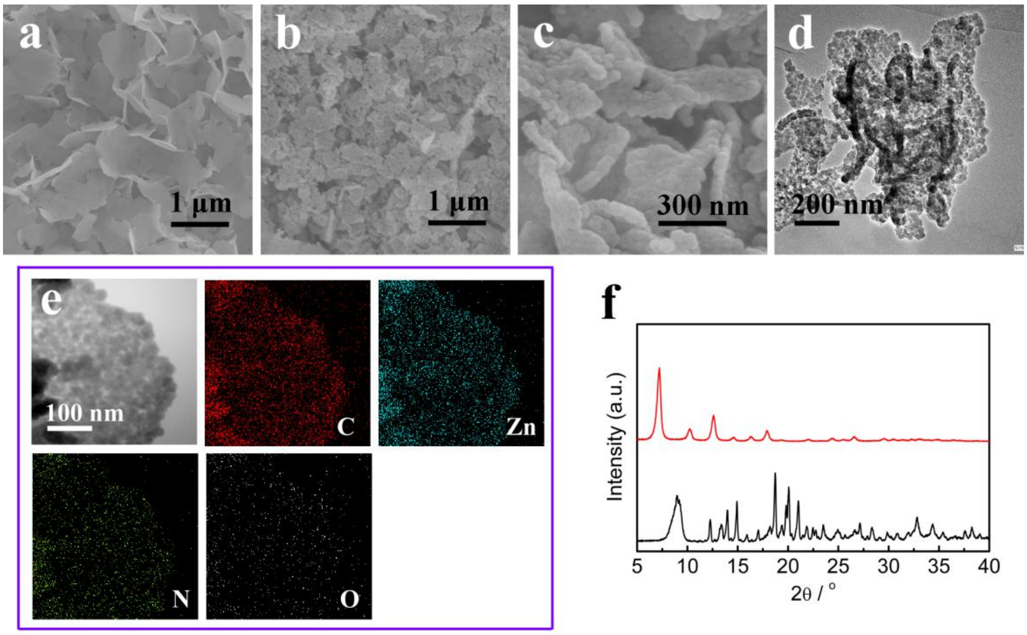

Supplementary Figure 19. SEM images of as-prepared Zn-HMT (a) and Zn-HMT-derived ZIF-8 nanosheets (b, c); TEM image of ZIF-8 nanosheets (d); EDX mapping images of ZIF-8 nanosheets (e); XRD patterns of materials before (black line) and after (red line) the reaction (f); the peaks of the red line all corresponded to ZIF-8 and no other peak was detected, indicating the transformation from Zn-HMT to ZIF-8.

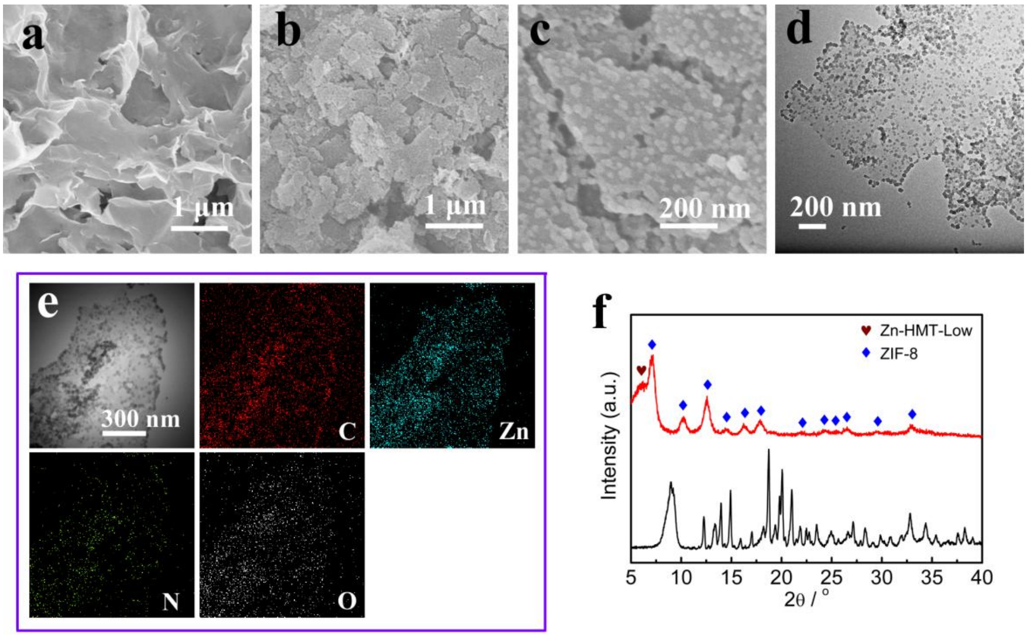

Supplementary Figure 20. SEM images of as-prepared Zn-HMT (a) and Zn-HMT-derived sesame pancake-like Zn-HMT@ZIF-8 nanosheets (b, c); TEM image of sesame pancake-like Zn-HMT@ZIF-8 nanosheets (d); EDX mapping images of sesame pancake-like Zn-HMT@ZIF-8 nanosheets (e); the XRD patterns of materials before (black line) and after (red line) the reaction (f), XRD results revealed the partial transformation from Zn-HMT to ZIF-8 (f), suggesting the sesame pancake-like structured MOF-5@ZIF-8 nanosheets. The edge part rather than the central part of each nanosheet was converted into ZIF-8 completely because more unsaturated coordination bonds lay at the edge of Zn-HMT nanosheets (c-e).

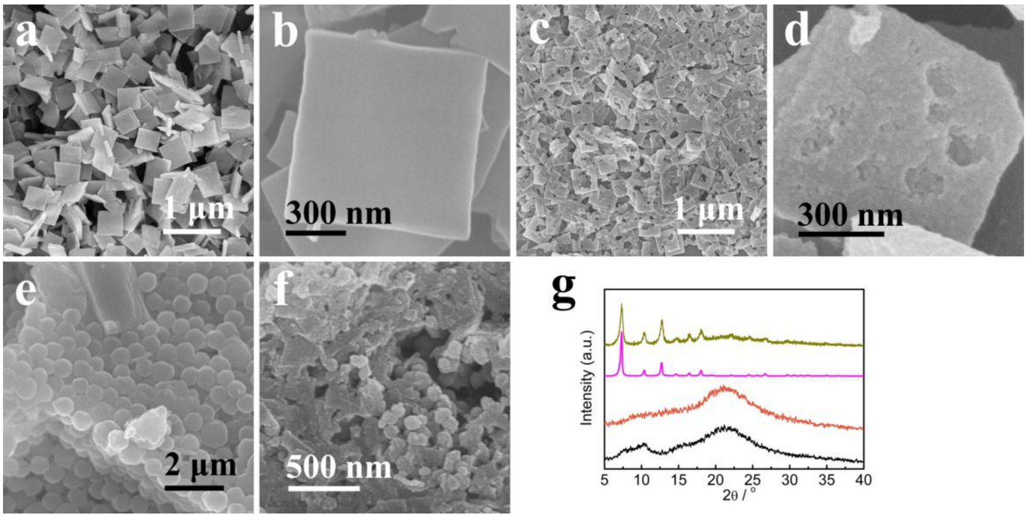

Supplementary Figure 21. SEM images of ZnCo-PPF-3 before (a, b) and after being placed in 1 M Hmim solution: ethanol as the solvent for 1 h reaction (c, d), ethanol as the solvent for 2 h reaction (e), ethanol/water (volume ratio $V_{ethanol}$:V_{water} =9:1) mixed solution for 1 h (f); XRD patterns of materials before and after the reaction (g): ZnCo-PPF-3 (black line), ethanol solution for 1 h (red line), ethanol solution for 2 h (magenta line), ethanol/water mixed solution for 1 h (dark yellow line). There were large holes in the center position of some nanosheets after ZnCo-PPF-3 nanosheets were placed in a 1 M Hmim ethanol solution for 1 h (c); the surface of the nanosheets became rougher and exhibited particle-like morphology features (d), but in its corresponding XRD pattern we could not observe any possible peak related to ZnCo-ZIF. Therefore, it could be concluded that the ligand exchange enabled the cleavage of the ZnCo-PPF-3 bonds but was not able to reassemble the topology structure of ZnCo-ZIF. This behavior was very different from the case of Zn-HMT due to the much more stable topological construction of ZnCo-PPF-3. In the ZnCo-PPF-3 nanosheets, one 5,10,15,20-tetrakis(4-carboxylphenyl)porphyrin ligand was metalated by one Zn/Co ion and linked by four $(Co/Zn)_2(COO)_4$ paddlewheel metal nodes to stack in an AB packing pattern (a “checkerboard-like” layered structure), which was further pillared by 4,4’-bipyridine to form the final structure with space group of *I4/mmm*[2]. By contrast, the two N atoms of HMT as a bidentate bridging ligand coordinated with Zn ions to be a zigzag chain; simultaneously, each chain was further hydrogen bonded by OH-N and OH-O interactions with

neighboring chains, giving rise to a 2D lamellar frameworks of Zn-HMT[3]. Since the metal ion coordination to organic ligands was much stronger than its coordination to water and hydrogen bonding interaction[4], Zn-HMT constructed by the hydrogen bonding between these zigzag chains was thus less stable than ZnCo-PPF-3 by metal-organic ligand coordination. If the reaction was prolonged to 2 h, the resulting product had a ZnCo-ZIF phase according to the XRD results; they were ~ 500 nm in microspheres and absolutely lost the initial morphology of the 2D nanosheet (e). If some H_2O was introduced to facilitate the fast nucleation of ZnCo-ZIF on the ZnCo-PPF-3 nanosheets, the product was a mixture of nanosheets and nanoparticles. The XRD peaks of ZnCo-ZIF might originate from the nanoparticles because the XRD pattern of unmixed nanosheets did not show ZIF peaks, and while even a very small amount of nanoparticles existed, the diffraction peaks of ZnCo-ZIF dominated.

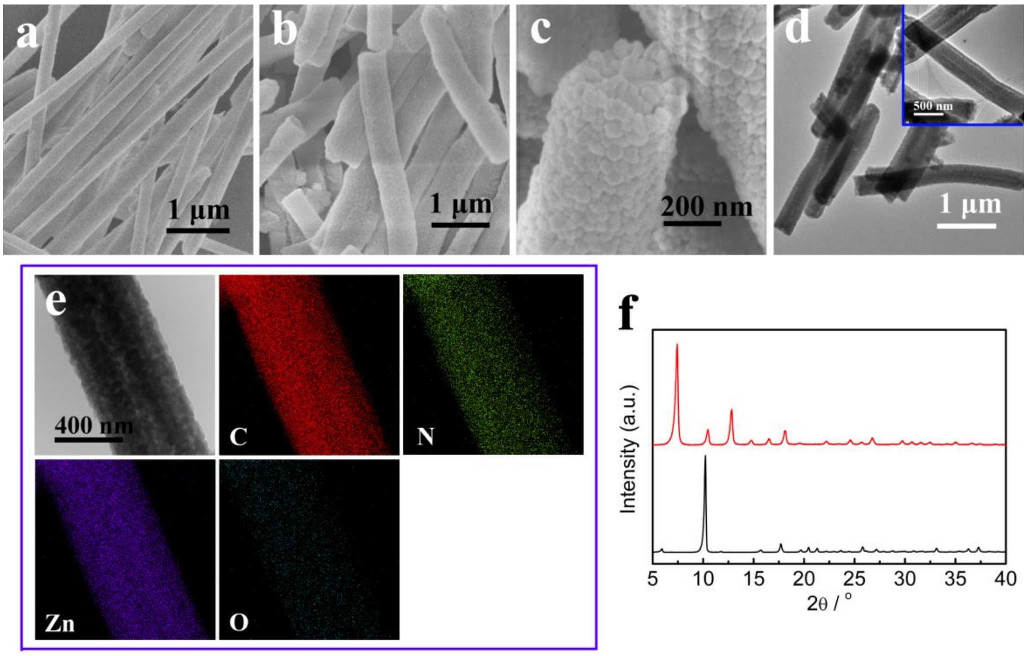

Supplementary Figure 22. SEM images of as-prepared Zn-BTC (a) and double-shelled ZIF-8 nanotubes (DT-ZIF-8) (b, c); TEM image of DT-ZIF-8 (d); EDX mapping images of DT-ZIF-8 (e); XRD patterns of materials before (black line) and after (red line) the reaction (f); the peaks of the red line all corresponded to ZIF-8 and no other peak was detected, indicating the transformation from Zn-BTC to ZIF-8. The inner shell was much thicker than the outer shell, which was very similar to the case of MOF-5-derived double-shelled hollow ZIF-8, and this phenomenon resulted from the restricted diffusion effect, which created a concentration gradient of mim^- ligand.

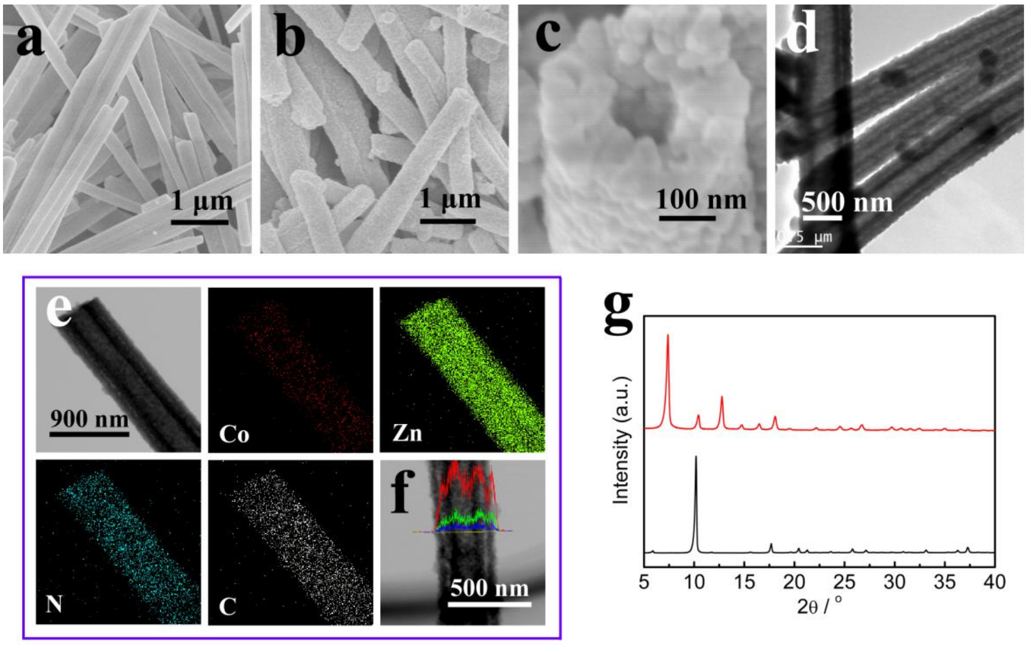

Supplementary Figure 23. SEM images of as-prepared 90Zn10Co-BTC (a) and its derived double-shelled ZnCo-ZIF nanotubes (DT-ZnCo-ZIF) (b, c); TEM image of DT-ZnCo-ZIF (d); EDX linear scanning images of DT-ZnCo-ZIF (e); XRD patterns of materials before (black line) and after (red line) the reaction (f); the peaks of the red line all corresponded to ZnCo-ZIF, and no other peak was detected, indicating the transformation from 90Zn10Co-BTC to ZnCo-ZIF. The inner shell was as thick as the outer shell which was very similar to the case of Co-doped MOF-5-derived double-shelled hollow ZnCo-ZIF. The Co^{2+} dissociated from 90Zn10Co-BTC retarded the ligand exchange process between BTC^{3-} and mim^{-}, and the rate of void generation was not as fast as that of Zn-BTC. As a result, the phase separation between mother MOF and daughter MOF was deferred, and the outer shell would become relatively thicker.

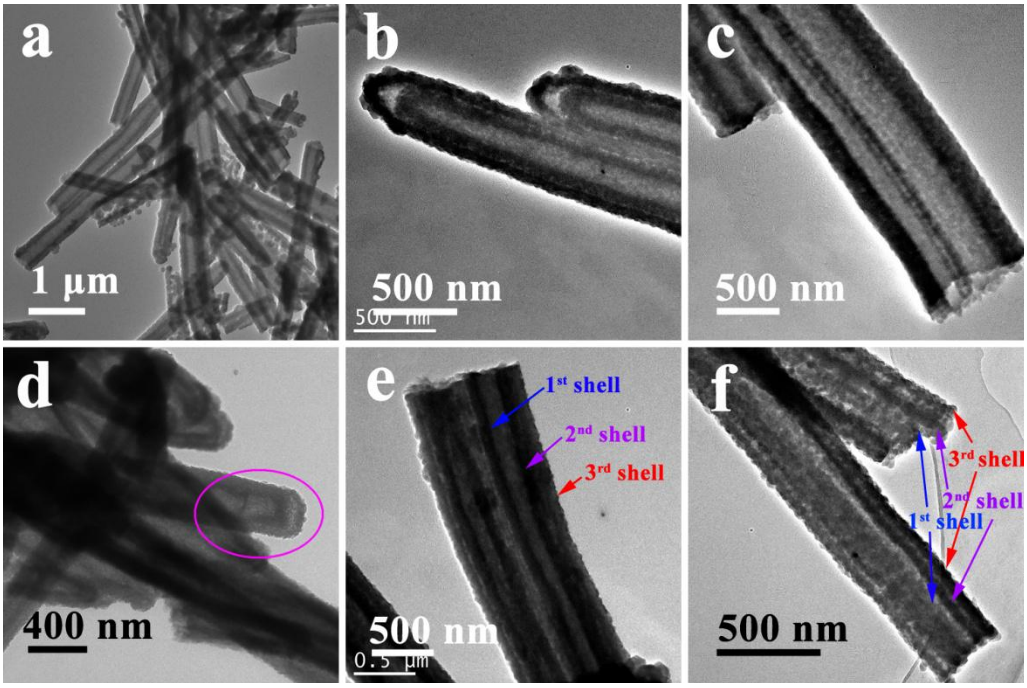

Supplementary Figure 24. TEM images of double-shelled (a-d) and triple-shelled ZnCo-ZIF nanotubes (e, f). Most of the products were normal double-shelled nanotubes with a narrow gap between the outer shell and inner shell (a, b); we could still observe some exceptional cases. For example, the diameter of the inner nanotube could be very small, which possibly resulted from the deferred phase separation of the mother MOF and daughter MOF, and the thickness of outer nanotube was much thicker than that of the inner nanotube (c). In addition, the trend of the triple-shelled structure could be seen at the tips of some nanotubes rather than in the middle due to the more effective ligand diffusion (d), and we indeed found very few triple-shelled nanotubes.

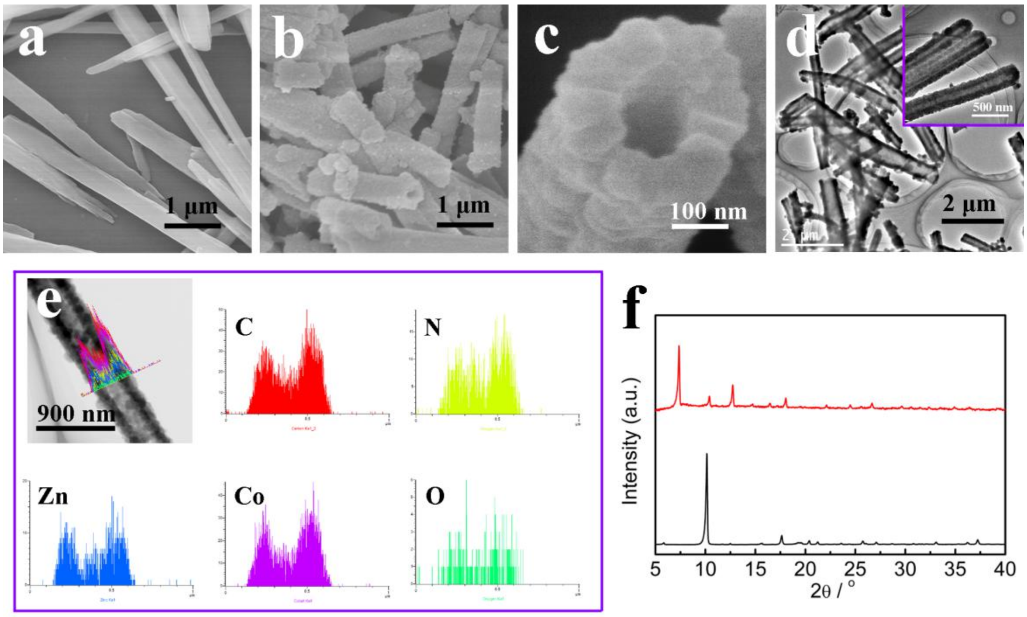

Supplementary Figure 25. SEM images of as-prepared 50Zn50Co-BTC (a) and its derived single-shelled ZnCo-ZIF nanotubes (ST-ZnCo-ZIF) (b, c); TEM image of ST-ZnCo-ZIF (d); EDX linear scanning images of ST-ZnCo-ZIF (e); XRD patterns of materials before (black line) and after (red line) the reaction (f); the peaks of the red line all corresponded to ZnCo-ZIF, and no other peak was detected, indicating the transformation from 50Zn50Co-BTC to ZnCo-ZIF.

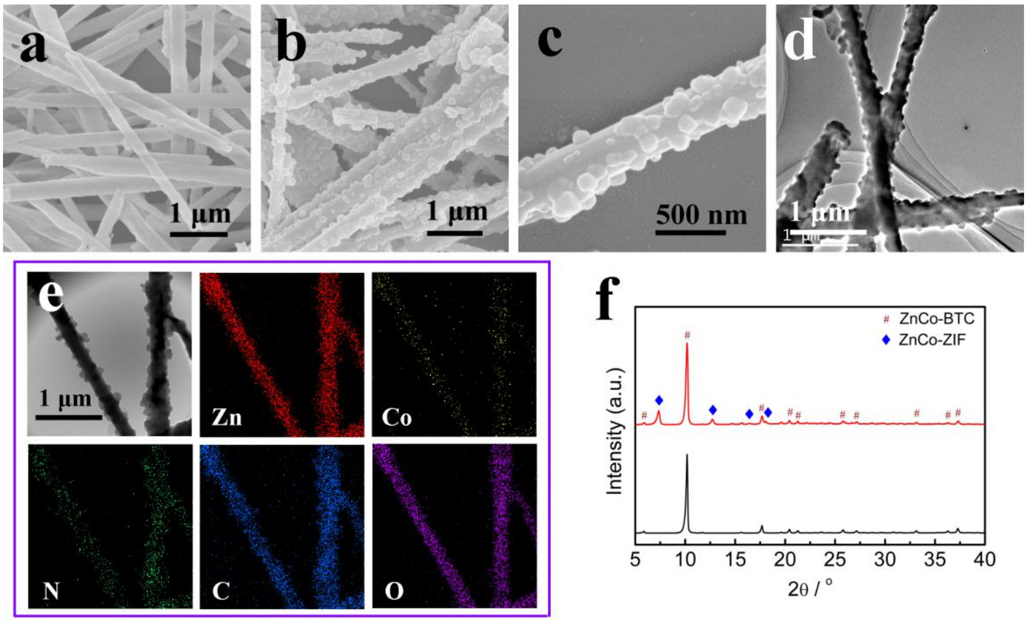

Supplementary Figure 26. SEM images of as-prepared 90Zn10Co-BTC (a) and its derived bead-on-string structured 90Zn10Co-BTC@ZnCo-ZIF (b, c); TEM image of bead-on-string structured 90Zn10Co-BTC@ZnCo-ZIF (d); EDX mapping images of bead-on-string structured 90Zn10Co-BTC@ZnCo-ZIF (e); XRD patterns of materials before (black line) and after (red line) the reaction (f); the XRD results revealed the partial transformation from 90Zn10Co-BTC to ZnCo-ZIF (f), suggesting the bead-on-string hybrid structure.

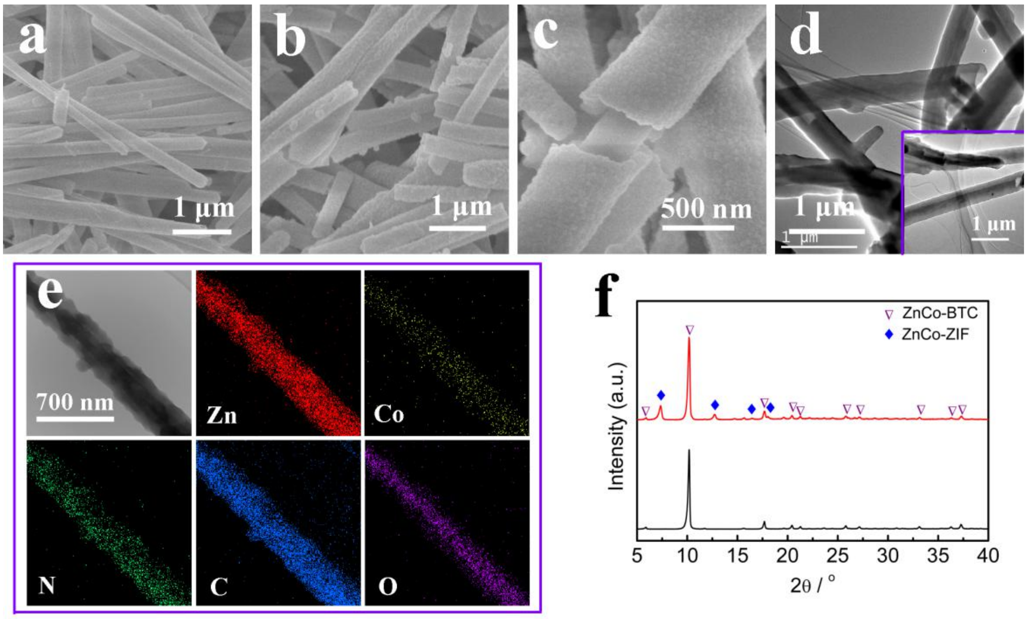

Supplementary Figure 27. SEM images of as-prepared 90Zn10Co-BTC (a) and its derived 90Zn10Co-BTC@ZnCo-ZIF core-shell structure (b, c); TEM image of 90Zn10Co-BTC@ZnCo-ZIF core-shell structure (d); EDX mapping images of 90Zn10Co-BTC@ZnCo-ZIF core-shell structure (e); XRD patterns of materials before (black line) and after (red line) the reaction (f); the XRD results revealed the partial transformation from 90Zn10Co-BTC to ZnCo-ZIF (f), suggesting the 90Zn10Co-BTC@ZnCo-ZIF core-shell hybrid structure.

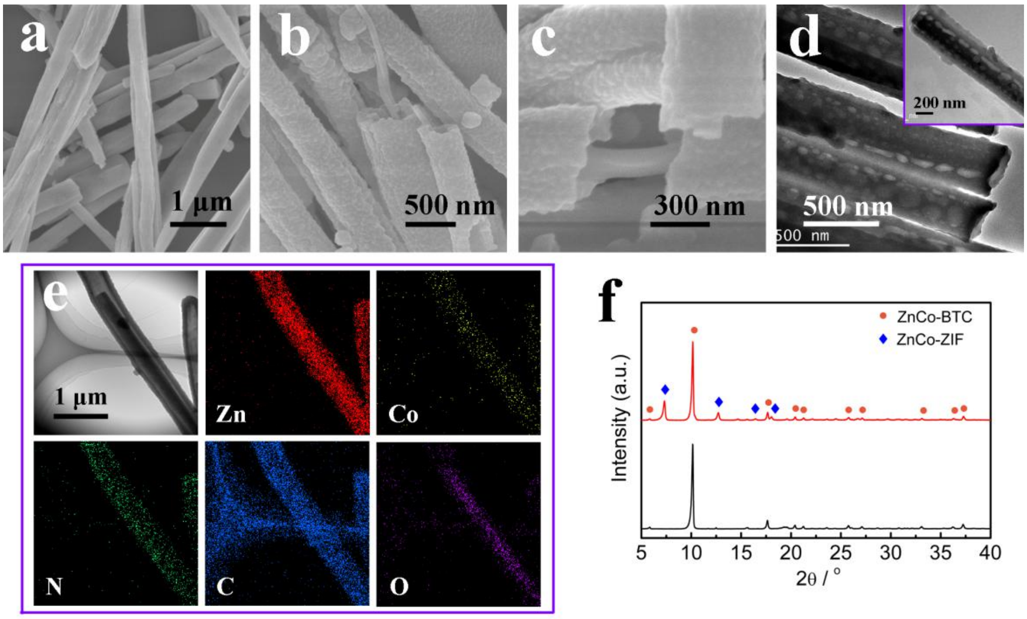

Supplementary Figure 28. SEM images of as-prepared 90Zn10Co-BTC (a) and its derived 90Zn10Co-BTC@ZnCo-ZIF nanowire-nanotube structure (b, c); TEM image of 90Zn10Co-BTC@ZnCo-ZIF nanowire-nanotube structure (d); EDX mapping images of 90Zn10Co-BTC@ZnCo-ZIF nanowire-nanotube structure (e); XRD patterns of materials before (black line) and after (red line) the reaction (f); the XRD results revealed the partial transformation from 90Zn10Co-BTC to ZnCo-ZIF (f), suggesting the 90Zn10Co-BTC@ZnCo-ZIF nanowire-nanotube hybrid structure. It was observed that some floccules were located between the ZnCo-ZIF nanotube and 90Zn10Co-BTC nanowire, which was assigned as the intermediate species in the crystallization of ZnCo-ZIF (d).

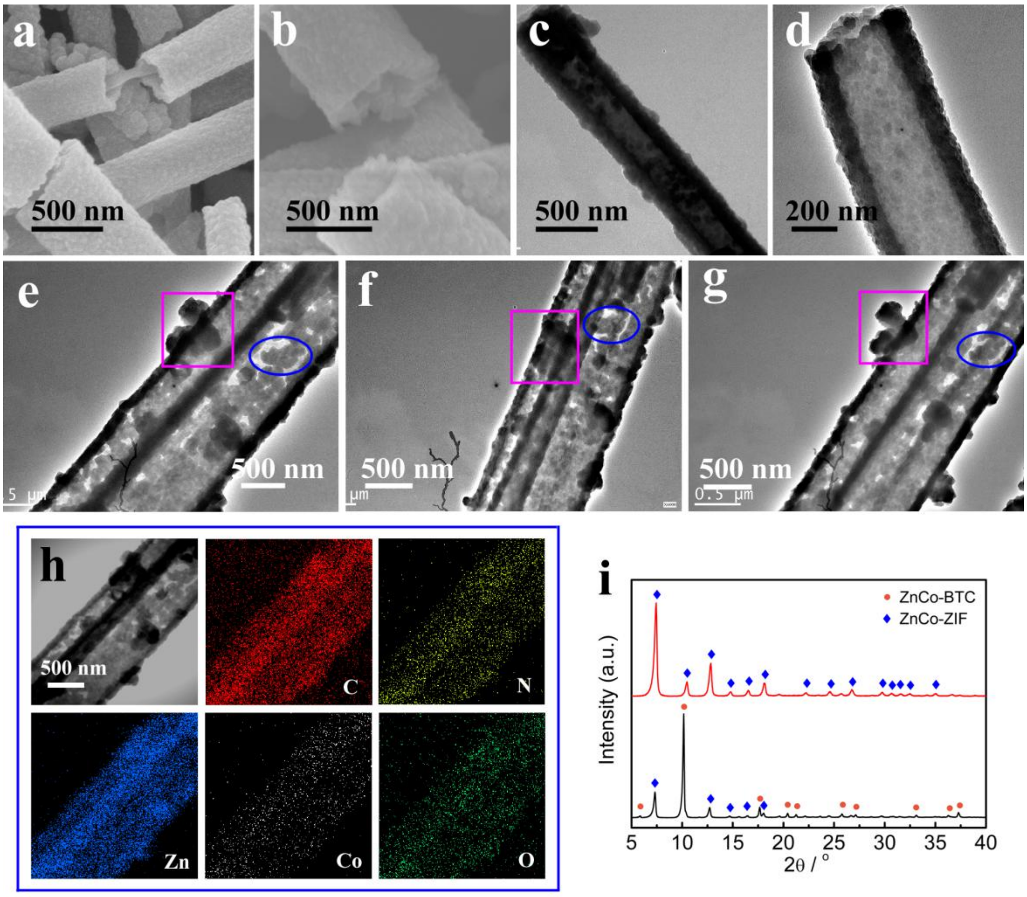

Supplementary Figure 29. SEM images of 90Zn10Co-BTC@ZnCo-ZIF nanowire-nanotube structure (a) and peapod-like ZnCo-ZIF (b); TEM images of peapod-like ZnCo-ZIF (c) and normal ST-ZnCo-ZIF (d); TEM images of peapod-like ZnCo-ZIF at 0° (e), 24° (f) and -24° (g); EDX mapping images of peapod-like ZnCo-ZIF (h); XRD patterns of materials before (black line) and after (red line) the reaction (i), suggesting the transformation of 90Zn10Co-BTC@ZnCo-ZIF nanowire-nanotube structure to peapod-like ZnCo-ZIF. Compared to normal ST-ZnCo-ZIF, the pea particles obviously had higher contrast than the pod tube (c); in addition, in the x-axis direction, based on the observations of clockwise rotation by 24° and counterclockwise rotation by 24° (e-g), pea particles (blue ellipse area) were located inside the nanotube all the time, and the relative location of particles outside the nanotube (pink rectangle area) changed along with the rotations.

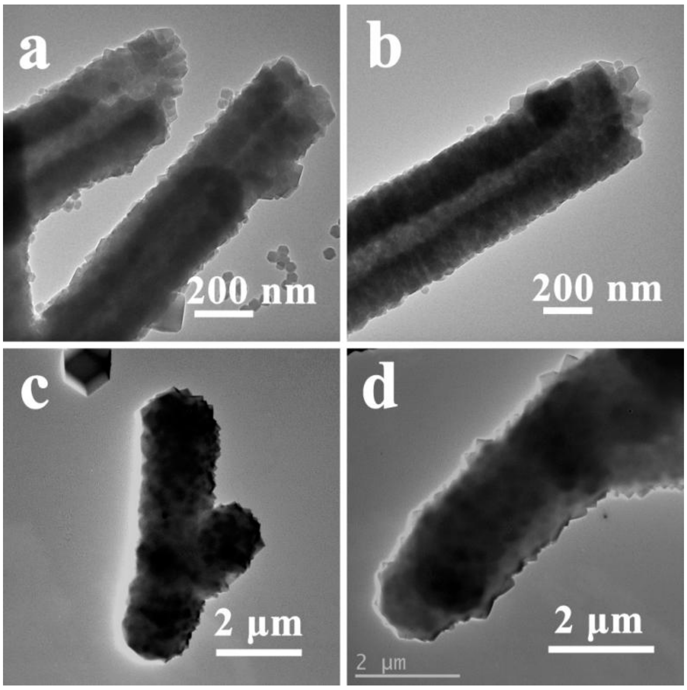

Supplementary Figure 30. TEM images of the final products by a traditional two-step synthesis. In this synthesis, ST-ZnCo-ZIF nanotubes were first synthesized starting from 90Zn10Co-BTC nanowires; in the second step, ST-ZnCo-ZIF nanotubes were immersed into ZnCo-ZIF precursor solution for several hours. We observed that ZnCo-ZIF preferred to grow on the inner and outer surface of tube walls (a) or that the open ends of ST-ZnCo-ZIF nanotubes were sealed by ZnCo-ZIF crystals (b). In addition, if the ST-ZnCo-ZIF nanotubes were broken into shorter nanotubes in an ultrasonic bath, the inner hollow space of all nanotubes was sealed, resulting in micron-scale wires (c, d).

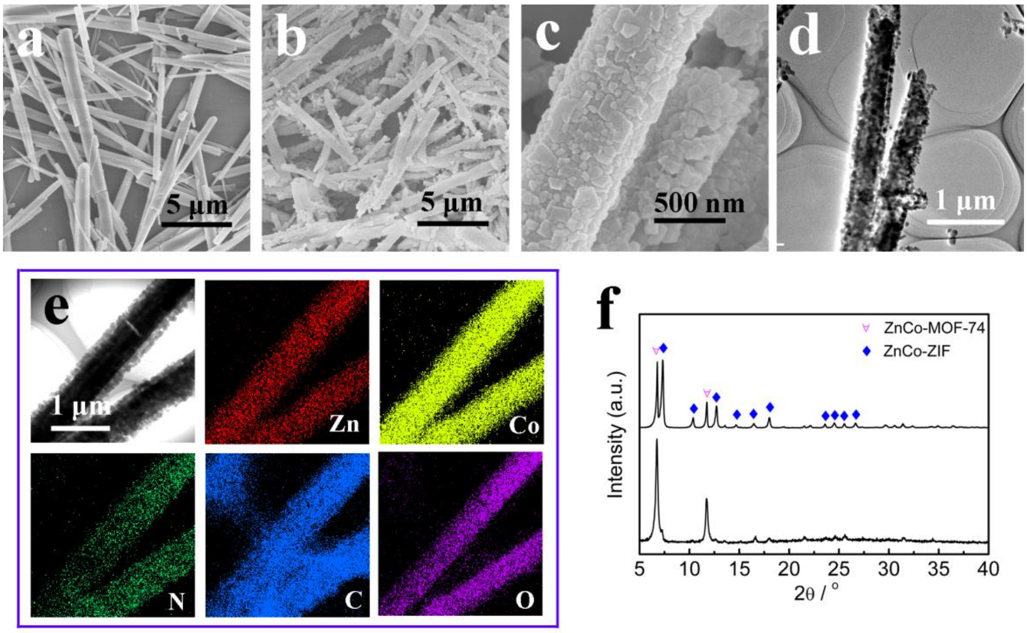

Supplementary Figure 31. SEM images of as-prepared 20Zn80Co-MOF-74 (a) and its derived 20Zn80Co-MOF-74@ZnCo-ZIF core-shell structure (b, c); TEM image of 20Zn80Co-MOF-74@ZnCo-ZIF core-shell structure (d); EDX mapping images of 20Zn80Co-MOF-74@ZnCo-ZIF core-shell structure (e); XRD patterns of materials before (black line) and after (red line) the reaction (f); the XRD results revealed the partial transformation from 20Zn80Co-MOF-74 to ZnCo-ZIF (f), suggesting the 20Zn80Co-MOF-74@ZnCo-ZIF core-shell hybrid structure.

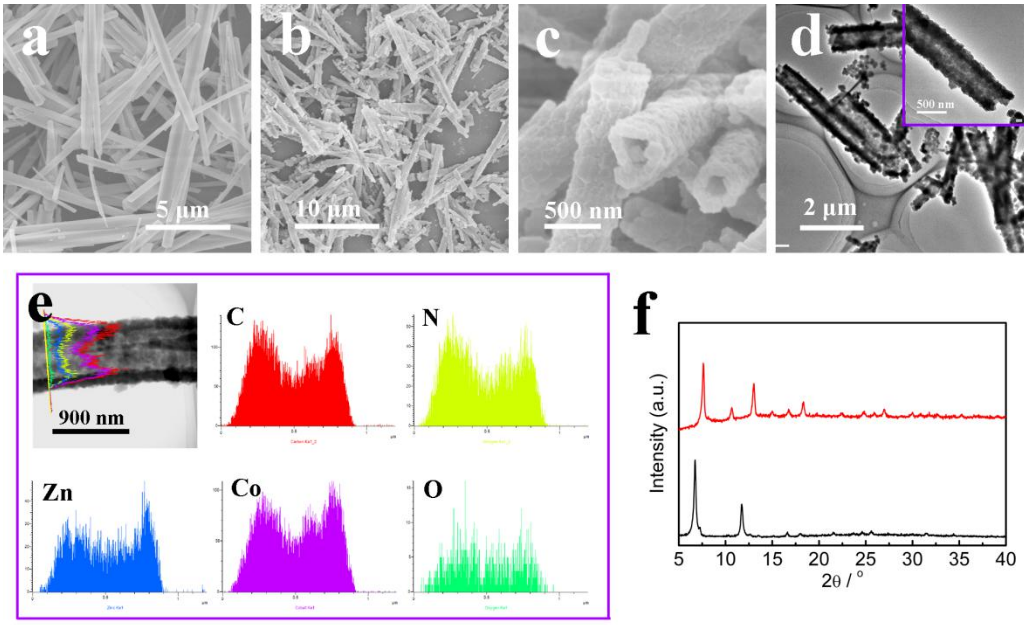

Supplementary Figure 32. SEM images of as-prepared 20Zn80Co-MOF-74 (a) and its derived single-shelled ZnCo-ZIF nanotubes (b, c); TEM image of 20Zn80Co-MOF-74-derived ZnCo-ZIF nanotubes (d); EDX linear scanning images of 20Zn80Co-MOF-74-derived ZnCo-ZIF nanotubes (e); XRD patterns of materials before (black line) and after (red line) the reaction (f); the peaks of the red line all corresponded to ZnCo-ZIF and no other peak was detected, indicating the transformation from 20Zn80Co-MOF-74 to ZnCo-ZIF. Because many ZnCo-ZIF nanoparticles formed in the synthesis, repeated centrifugation procedures were needed to remove the by-products. We could see that the preparation of ZnCo-ZIF nanotube from ZnCo-BTC required much milder reaction conditions than that from 20Zn80Co-MOF-74, which resulted from the weak hydrogen bond involved in the construction of ZnCo-BTC framework. The ZnCo-BTC framework was composed of zigzag chains of hydrogen-bonded tetra-aqua Zn/Co benzenetricarboxylate[4]. In contrast, the helical Zn/Co-O-C rods of ZnCo-MOF-74 were assembled by edge-sharing octahedral coordination, and the rods were linked by the benzene units of the 2,5-dihydroxy-1,4-benzenedicarboxylic acid to produce bnn-type parallel rod packing[5]. Because both metal-H_2O and hydrogen bonds were weaker interaction than metal-organic ligand coordination, therefore, ZnCo-MOF-74 was more stable than ZnCo-BTC, resulting in the harder conversion to ZnCo-ZIF.

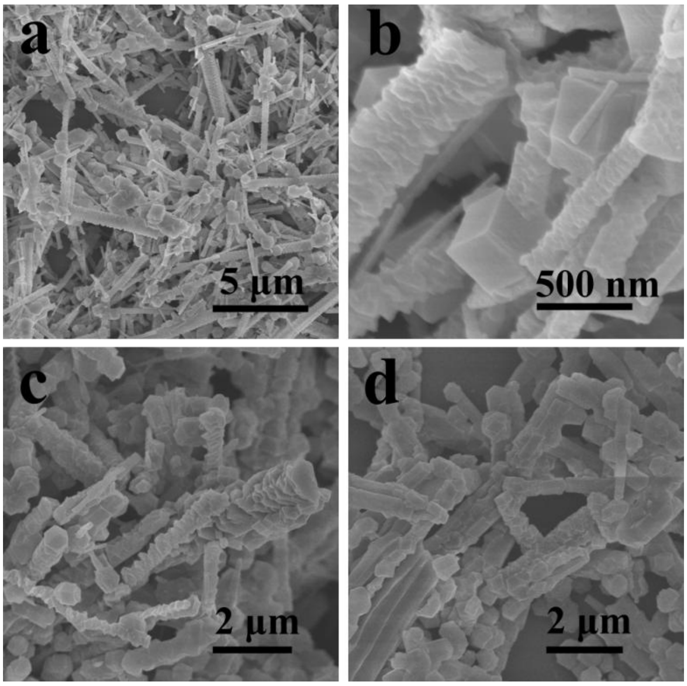

Supplementary Figure 33. SEM images of 10Zn90Co-MOF-74 nanowires in the presence of 1.2 M (a, b) and 2.4 M (c, d) Hmim ethanol/water (volume ratio of $V_{ethanol:}V_{water}$=1:1) mixed solution at 70 °C for 15 min. With the increase of Co content from 80% to 90%, the particle size of ZnCo-ZIF became larger and there was no nanotube structure but nanowires; in addition, excess particle-like by-products were found in the final product even after centrifugation at a low speed several times, and more Co content caused the loss of morphology control.

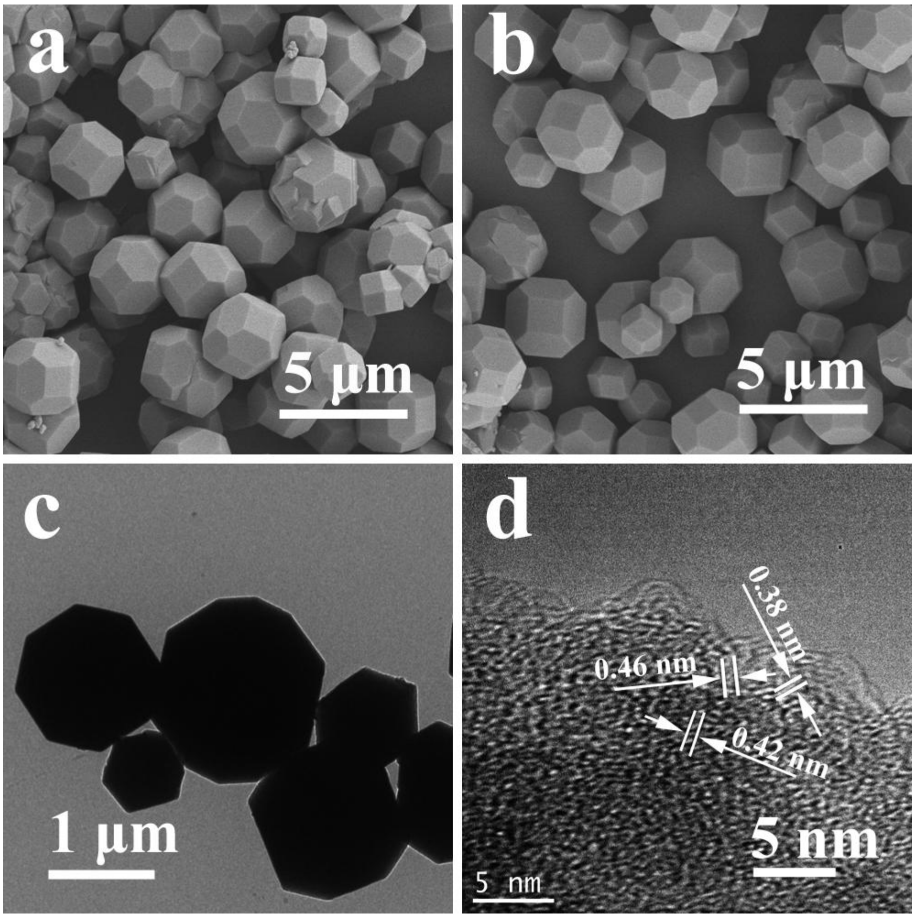

Supplementary Figure 34. SEM images of ZIF-8 particles before (a) and after (b) the carbonization treatment; TEM image (c) and corresponding high-resolution TEM image (d) of ZIF-8-derived nanoporous carbon particles.

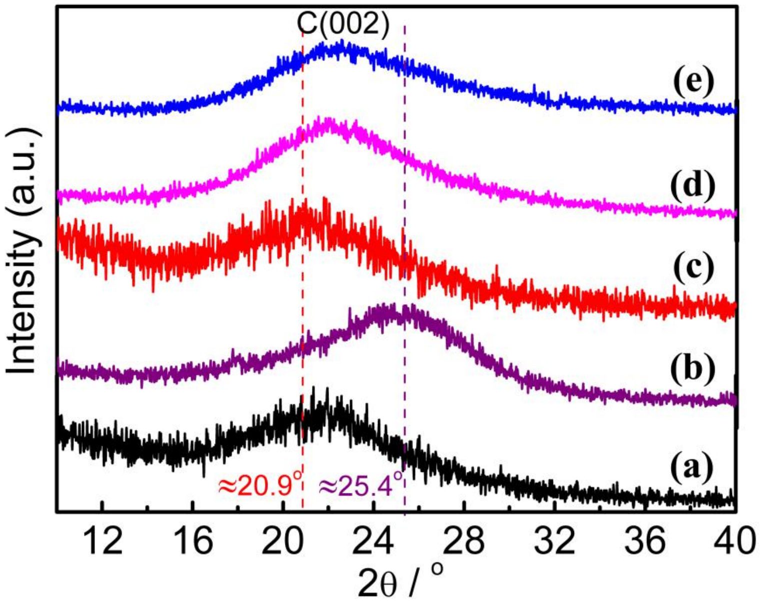

Supplementary Figure 35. XRD patterns of s-Zn-ZIF-C (a), DH-Zn-ZIF-C (b), NS-Zn-ZIF-C (c), DT-Zn-ZIF-C (d) and DT-ZnCo-ZIF-C (e). The C(002) diffraction peak of DH-Zn-ZIF-C and NS-Zn-ZIF-C was located at the right-most (~ 25.4^{o}) and left-most (~ 20.9^{o}) positions, respectively, implying that DH-Zn-ZIF-C had the smallest interplanar spacing of C(002) and that NS-Zn-ZIF-C had the largest interplanar spacing value.

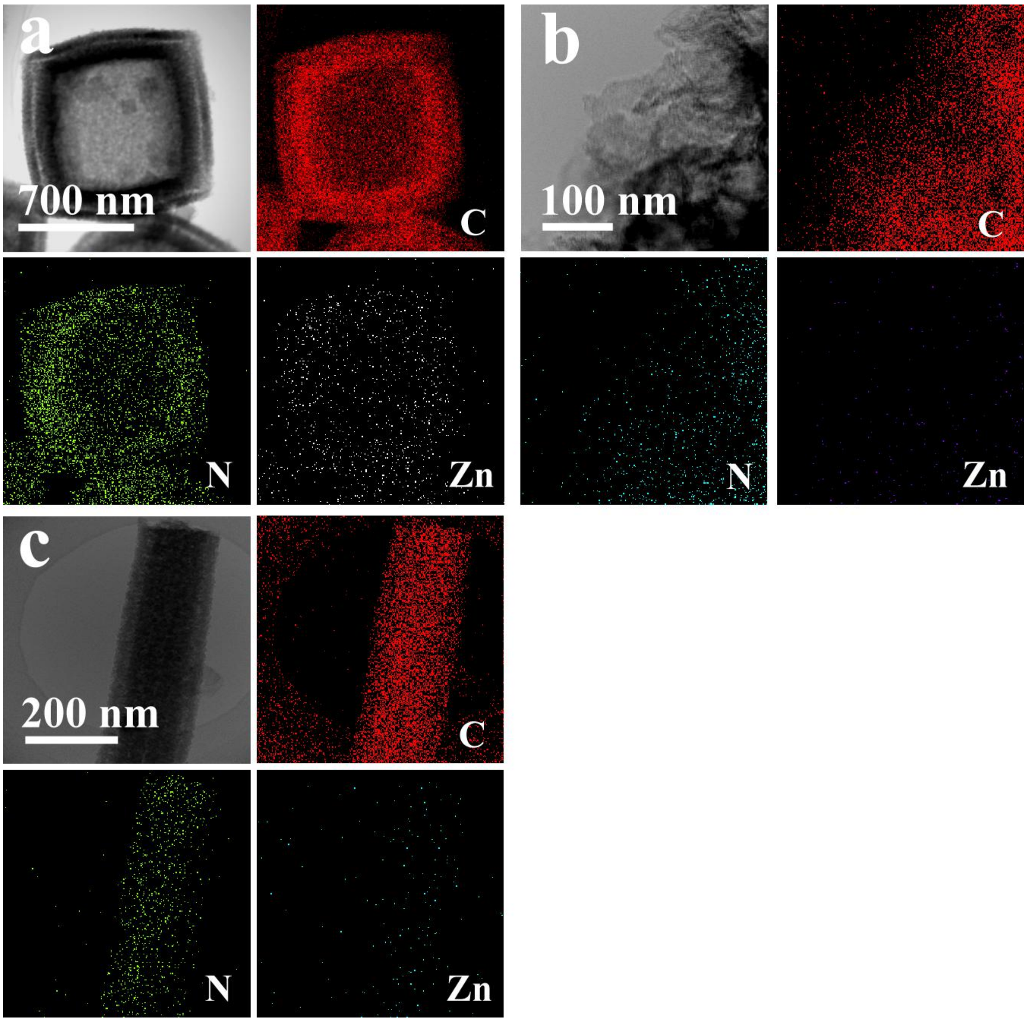

Supplementary Figure 36. TEM-EDX mapping images of DH-Zn-ZIF-C (a), NS-Zn-ZIF-C (b) and DT-Zn-ZIF-C (c).

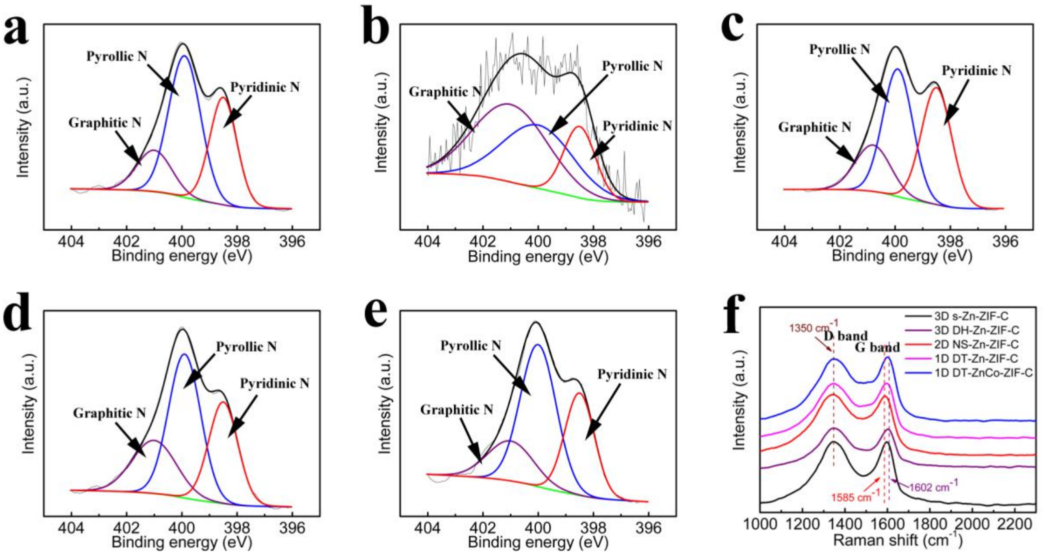

Supplementary Figure 37. The high-resolution N 1s XPS spectra of s-Zn-ZIF-C (a), DH-Zn-ZIF-C (b), NS-Zn-ZIF-C (c), DT-Zn-ZIF-C (d) and DT-ZnCo-ZIF-C (e); the Raman spectra of solid ZIF-8 particle-derived carbon (s-Zn-ZIF-C), DH-Zn-ZIF-C, NS-Zn-ZIF-C, DT-Zn-ZIF-C and DT-ZnCo-ZIF-C. The high-resolution N 1s spectra of s-Zn-ZIF-C, DH-Zn-ZIF-C, NS-Zn-ZIF-C, DT-Zn-ZIF-C and DT-ZnCo-ZIF-C were fitted with three peaks at approximately 398.5, 399.9 and 400.8 eV, corresponding to pyridinic N, pyrrolic N and graphitic N, respectively. The results revealed the successful N-doping of all ZIF-derived nanoporous carbon, which would benefit the excellent Na^+ storage performance. In the Raman spectra, the G band of NS-Zn-ZIF-C shifted to the left-most positions (~ 1585 cm^{-1}), which indicated the most n-type doping on nanoporous carbon, and the G band of DH-Zn-ZIF-C shifted to the right-most (~ 1602 cm^{-1}) positions, in agreement with the XPS and XRD results.

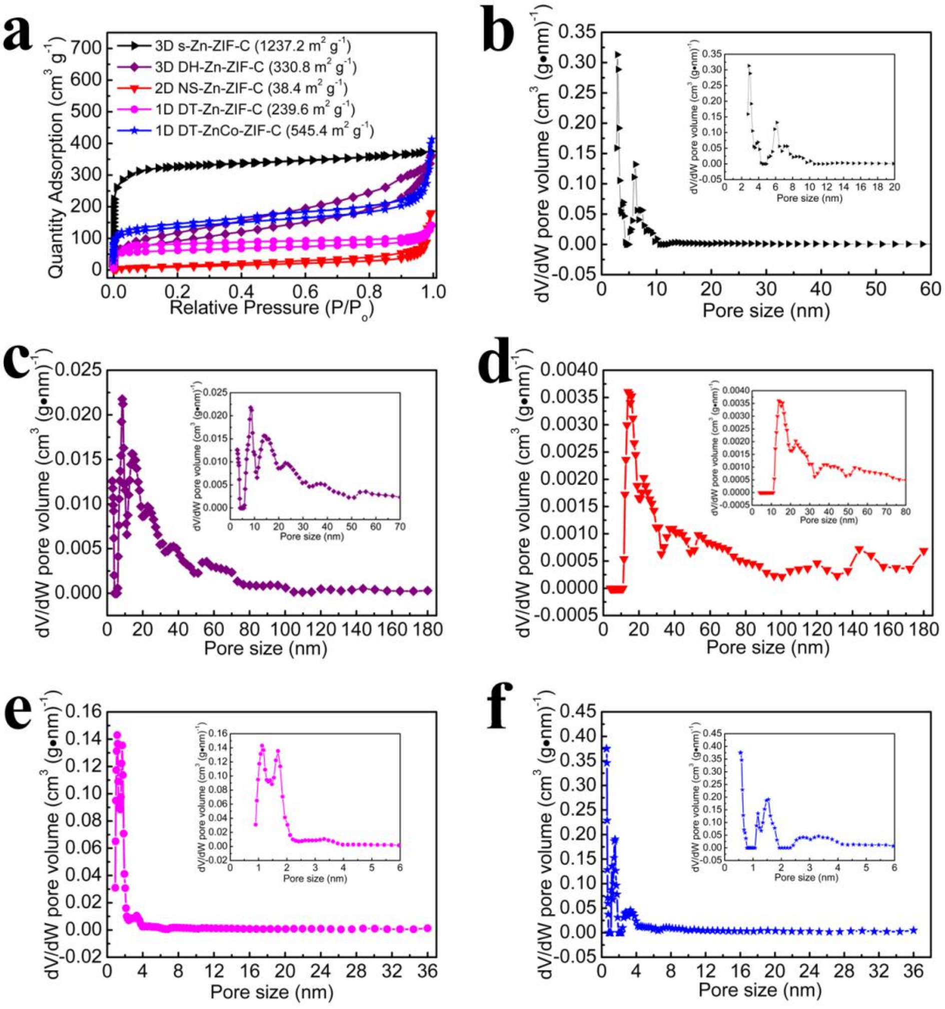

Supplementary Figure 38. Nitrogen adsorption-desorption isotherms of ZIF-derived porous carbon (a), the Barrett-Joyner-Halenda pore size distribution plots of s-Zn-ZIF-C (b), DH-Zn-ZIF-C (c), NS-Zn-ZIF-C (d), DT-Zn-ZIF-C (e) and DT-ZnCo-ZIF-C (f).

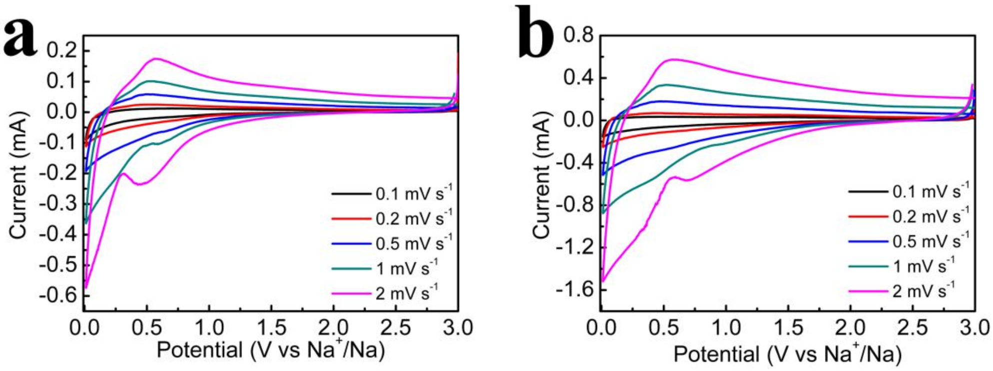

Supplementary Figure 39. CV plots of s-Zn-ZIF-C (a) and DT-Zn-ZIF-C (b) at different scan rates.

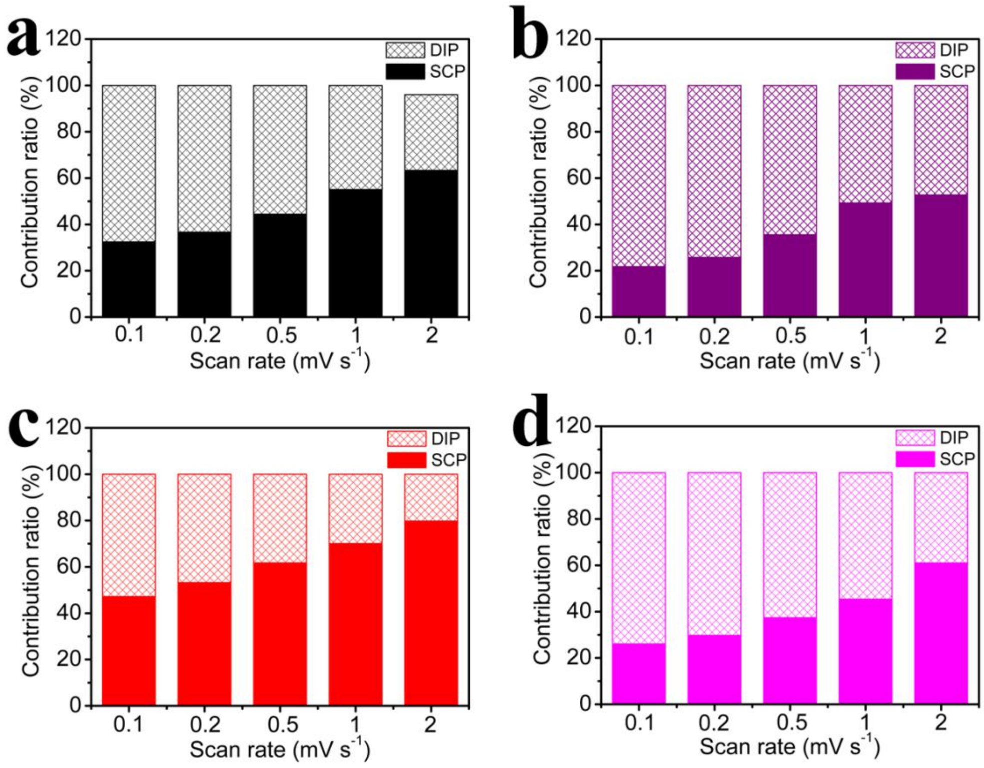

Supplementary Figure 40. Contribution ratio of DIP and SCP versus scan rate calculated from CV curves of 3D s-Zn-ZIF-C (a), 3D DH-Zn-ZIF-C (b), 2D NS-Zn-ZIF-C (c) and 1D DT-Zn-ZIF-C (d) from Figure 4 d-e and Supplementary Figure 39.

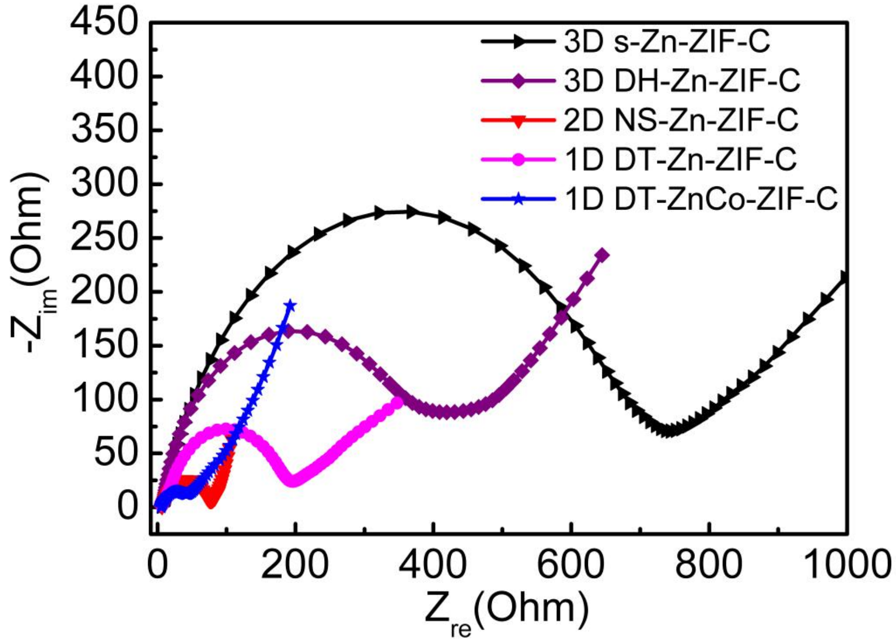

Supplementary Figure 41. Nyquist plots of s-Zn-ZIF-C, DH-Zn-ZIF-C, NS-Zn-ZIF-C, DT-Zn-ZIF-C and DT-ZnCo-ZIF-C at a range of 0.1 Hz ~100 kHz.

Supplementary Table 1. Overall N-doping content of ZIF-derived porous carbon and the specific content of pyridinic N, pyrrolic N and graphitic N extracted from Supplementary Figure 37a-e.

Materials	Overall N	Pyridinic N	Pyrrolic N	Graphitic N
**s-Zn-ZIF-C**	11.11%	3.70%	5.62%	1.79%
**DH-Zn-ZIF-C**	3.69%	0.67%	1.32%	1.70%
**NS-Zn-ZIF-C**	13.34%	5.24%	5.66%	2.44%
**DT-Zn-ZIF-C**	9.30%	2.80%	4.31%	2.19%
**DT-ZnCo-ZIF-C**	8.47%	2.58%	4.33%	1.56%

Supplementary Table 2. Na^+ storage properties of MOF-derived carbon materials in recent literature.

Samples	Specific Capacity	Rate Capability	Cycling Stability	References
Zn-hexamine-derived N-doped carbon nanosheets	317 mAh g^{-1} at 100 mA g^{-1}	235 mAh g^{-1} at 2 A g^{-1} ; 194 mAh g^{-1} at 10 A g^{-1};	76.9% after 1000 cycles at 5 A g^{-1}	3
MOF-5-derived porous carbon cubes	233.4 mAh g^{-1} at 100 mA g^{-1}	110 mAh g^{-1} at 0.8 A g^{-1}; 100 mAh g^{-1} at 3.2 A g^{-1};	~ 100% after 5000 cycles at 3.2 A g^{-1}	6
ZIF-8-derived hollow carbon nanobubbles	240 mAh g^{-1} at 50 mA g^{-1}	90 mAh g^{-1} at 15 A g^{-1}	~ 100% after 1000 cycles at 10 A g^{-1}	7
Mn-MOF-derived hollow porous carbon microspheres	313.8 mAh g^{-1} at 100 mA g^{-1}	198.3 mAh g^{-1} at 1.6 A g^{-1}; 115.8 mAh g^{-1} at 5 A g^{-1};	~ 100% after 100 cycles at 0.1 A g^{-1}	8
ZnCo-ZIF-derived N-doped carbon nanotubes	346 mAh g^{-1} at 120 mA g^{-1}	238 mAh g^{-1} at 1.5 A g^{-1};128 mAh g^{-1} at 7 A g^{-1}	~ 100% after 10000 cycles at 4.5 A g^{-1}	9
Cu-hexamine-derived N-doped carbon	303 mAh g^{-1} at 100 mA g^{-1}	160 mAh g^{-1} at 2 A g^{-1}; 142 mAh g^{-1} at 5 A g^{-1}	86.6% after 500 cycles at 5 A g^{-1}	10
ZIF-67-derived hierarchical carbon	220 mAh g^{-1} at 100 mA g^{-1}	-----	~100% after 500 cycles at 100 mA g^{-1}	11
Mn-MOF-derived N-doped wrinkled carbon foils	306 mAh g^{-1} at 50 mA g^{-1}	165 mAh g^{-1} at 8 A g^{-1};150 mAh g^{-1} at 10 A g^{-1}	72.8% after 1000 cycles at 1 A g^{-1}	12

MIL-100-derived S and N co-doped shell-like carbon	448 mAh g^{-1} at 100 mA g^{-1}	337 mAh g^{-1} at 10 A g^{-1}; 169 mAh g^{-1} at 32 A g^{-1}	97.8% after 4500 cycles at 16 A g^{-1}	13
1D double-shelled ZnCo-ZIF-derived nanoporous carbon nanotubes	455 mAh g^{-1} at 100 mA g^{-1}	305 mAh g^{-1} at 2 A g^{-1};178 mAh g^{-1} at 10 A g^{-1}	96.6% after 3000 cycles at 2 A g^{-1}	This work

Supplementary Table 3. Na^+ storage properties of other carbon materials in recent literature.

Samples	Specific Capacity	Rate Capability	Cycling Stability	References
Expanded graphite	284 mAh g^{-1} at 20 mA g^{-1}	184 mAh g^{-1} at 0.1 A g^{-1}; 91 mAh g^{-1} at 0.2 A g^{-1}	99.9% after 2000 cycles at 100 mA g^{-1}	14
Carbon quantum dots	290 mAh g^{-1} at 200 mA g^{-1}	166 mAh g^{-1} at 2A g^{-1}; 104 mAh g^{-1} at 10 A g^{-1}	105.4% after 5000 cycles at 5 A g^{-1}	15
N-doped carbon nanofiber films	377 mAh g^{-1} at 100 mA g^{-1}	145 mAh g^{-1} at 15 A g^{-1}	99% after 7000 cycles at 5 A g^{-1}	16
3D amorphous carbon	205 mAh g^{-1} at 300 mA g^{-1}	66 mAh g^{-1} at 9.6 A g^{-1}	91.7% after 600 cycles at 0.3 A g^{-1}	17
N-doped hard carbon nanoshells	325 mAh g^{-1} at 100 mA g^{-1}	63 mAh g^{-1} at 5 A g^{-1}	53.5% after 200 cycles at 0.1 A g^{-1}	18
S-doped graphene	436 mAh g^{-1} at 50 mA g^{-1}	217.1 mAh g^{-1} at 3.2 A g^{-1}	100% after 1000 cycles at 2 A g^{-1}	19
S, N, P-doped hierarchical vesicular carbon	366.5 mAh g^{-1} at 100 mA g^{-1}	142.6 mAh g^{-1} at 5 A g^{-1}	---	20
g-C_3N_4-derived N-rich graphene	264 mAh g^{-1} at 100 mA g^{-1}	148.5 mAh g^{-1} at 5 A g^{-1}; 56.6 mAh g^{-1} at 40 A g^{-1}	98.6% after 2000 cycles at 0.5 A g^{-1}	21

Honeycomb-like N-rich hierarchically porous carbon	371.4 mAh g^{-1} at 30 mA g^{-1}	150 mAh g^{-1} at 2 A g^{-1}	91.4% after 3000 cycles at 0.5 A g^{-1}	22
Defect-rich soft carbon porous nanosheets	232.2 mAh g^{-1} at 20 mA g^{-1}	103.8 mAh g^{-1}at 1 A g^{-1}	93% after 3500 cycles at 0.8 A g^{-1}	23
1D double-shelled ZnCo-ZIF-derived nanoporous carbon nanotubes	455 mAh g^{-1} at 100 mA g^{-1}	305 mAh g^{-1} at 2 A g^{-1};178 mAh g^{-1} at 10 A g^{-1}	96.6% after 3000 cycles at 2 A g^{-1}	This work

Supplementary references

1. Xu, X. et al. Synthesis of ZIF-8 hollow spheres via MOF-to-MOF conversion. *ChemistrySelect* **1**, 1763-1767 (2016).
2. Cao, F. et al. Synthesis of two-dimensional $CoS_{1.097}$/nitrogen-doped carbon nanocomposites using metal-organic framework nanosheets as precursors for supercapacitor application. *J. Am. Chem. Soc.* **138**, 6924-6927 (2016).
3. Liu, S., Zhou, J. & Song, H. 2D Zn-hexamine coordination frameworks and their derived N-rich porous carbon nanosheets for ultrafast sodium storage. *Adv. Energy Mater.* **8**, 1800569 (2018).
4. Yaghi, O.M., Li, H. & Groy, T.L. Construction of porous solids from hydrogen-bonded metal complexes of 1,3,5-benzenetricarboxylic acid. *J. Am. Chem. Soc.* **118**, 9096-9101 (1996).
5. Rosi, N.L. et al. Rod packings and metal-organic frameworks constructed from rod-shaped secondary building units. *J. Am. Chem. Soc.* **127**, 1504-1518 (2005).
6. Zou, G. et al. Cube-shaped porous carbon derived from MOF-5 as advanced material for sodium-ion batteries. *Electrochim. Acta* **196**, 413-421 (2016).
7. Zhang, W. et al. Hollow carbon nanobubbles: monocrystalline MOF nanobubbles and their pyrolysis. *Chem. Sci.* **8**, 3538-3546 (2017).
8. Zou, G. et al. 3D hollow porous carbon microspheres derived from Mn-MOFs and their electrochemical behavior for sodium storage. *J. Mater. Chem. A* **5**, 23550-23558 (2017).
9. Chen, Y. et al. Nitrogen-doped carbon for sodium-ion battery anode by self-etching and graphitization of bimetallic MOF-based composite. *Chem* **3**, 152-163 (2017).
10. Liu, S., Zhou, J. & Song, H. Tailoring highly N-doped carbon materials from hexamine-based MOFs: superior performance and new insight into the roles of N configurations in Na-ion storage. *Small* **14**, e1703548 (2018).
11. Du, M. et al. Stereoselectively assembled metal-organic framework (MOF) host for catalytic synthesis of carbon hybrids for alkaline-metal-ion batteries. *Angew. Chem. Int. Ed.* **58**, 5307-5311 (2019).
12. Kong, L., Zhu, J., Shuang, W. & Bu, X.-H. Nitrogen-doped wrinkled carbon foils derived from MOF nanosheets for superior sodium storage. *Adv. Energy Mater.* **8**, 1801515 (2018).
13. Mahmood, A. et al. Ultrafast sodium/potassium-ion intercalation into hierarchically porous thin carbon shells. *Adv. Mater.* **31**, e1805430 (2019).

14. Wen, Y. et al. Expanded graphite as superior anode for sodium-ion batteries. *Nat. Commun.* **5**, 4033 (2014).

15. Hou, H., Banks, C.E., Jing, M., Zhang, Y. & Ji, X. Carbon quantum dots and their derivative 3D porous carbon frameworks for sodium-ion batteries with ultralong cycle life. *Adv. Mater.* **27**, 7861-7866 (2015).

16. Wang, S. et al. Free-standing nitrogen-doped carbon nanofiber films: integrated electrodes for sodium-ion batteries with ultralong cycle life and superior rate capability. *Adv. Energy Mater.* **6**, 1502217 (2016).

17. Lu, P., Sun, Y., Xiang, H., Liang, X. & Yu, Y. 3D amorphous carbon with controlled porous and disordered structures as a high-rate anode material for sodium-ion batteries. *Adv. Energy Mater.* **8**, 1702434 (2018).

18. Huang, S. et al. N-doping and defective nanographitic domain coupled hard carbon nanoshells for high performance lithium/sodium storage. *Adv. Funct. Mater.* **28**, 1706294 (2018).

19. Quan, B. et al. Solvothermal-derived S-doped graphene as an anode material for sodium-ion batteries. *Adv. Sci.* **5**, 1700880 (2018).

20. Zou, G. et al. Advanced hierarchical vesicular carbon Co-doped with S, P, N for high-rate sodium storage. *Adv. Sci.* **5**, 1800241 (2018).

21. Liu, J. et al. Graphitic carbon nitride (g-C_3N_4)-derived N-rich graphene with tuneable interlayer distance as a high-rate anode for sodium-ion batteries. *Adv. Mater.* **31**, 1901261 (2019).

22. Hu, X. et al. Nitrogen-rich hierarchically porous carbon as a high-rate anode material with ultra-stable cyclability and high capacity for capacitive sodium-ion batteries. *Nano Energy* **56**, 828-839 (2019).

23. Yao, X. et al. Defect-rich soft carbon porous nanosheets for fast and high-capacity sodium-ion storage. *Adv. Energy Mater.* **9**, 1803260 (2018).
